# Ultradeep Microbial Communities at 4.4 km within Crystalline Bedrock: Implications for Habitability in a Planetary Context

**DOI:** 10.3390/life10010002

**Published:** 2020-01-04

**Authors:** Lotta Purkamo, Riikka Kietäväinen, Maija Nuppunen-Puputti, Malin Bomberg, Claire Cousins

**Affiliations:** 1 School of Earth and Environmental Sciences, University of St Andrews, St Andrews KY16 9AL, UK; 2 Geological Survey of Finland, 02151 Espoo, Finland; 3 Department of Geosciences and Geography, University of Helsinki, 00014 Helsinki, Finland; 4 VTT Technical Research Centre of Finland, 02044 VTT, Finland

**Keywords:** deep subsurface, deep biosphere, bedrock, microbial ecology, extraterrestrial habitat analog, environmental microbiology, microbial community, low biomass

## Abstract

The deep bedrock surroundings are an analog for extraterrestrial habitats for life. In this study, we investigated microbial life within anoxic ultradeep boreholes in Precambrian bedrock, including the adaptation to environmental conditions and lifestyle of these organisms. Samples were collected from Pyhäsalmi mine environment in central Finland and from geothermal drilling wells in Otaniemi, Espoo, in southern Finland. Microbial communities inhabiting the up to 4.4 km deep bedrock were characterized with phylogenetic marker gene (16S rRNA genes and fungal ITS region) amplicon and DNA and cDNA metagenomic sequencing. Functional marker genes (*dsr*B, *mcr*A, *nar*G) were quantified with qPCR. Results showed that although crystalline bedrock provides very limited substrates for life, the microbial communities are diverse. Gammaproteobacterial phylotypes were most dominant in both studied sites. *Alkanindiges* -affiliating OTU was dominating in Pyhäsalmi fluids, while different depths of Otaniemi samples were dominated by *Pseudomonas*. One of the most common OTUs detected from Otaniemi could only be classified to phylum level, highlighting the uncharacterized nature of the deep biosphere in bedrock. Chemoheterotrophy, fermentation and nitrogen cycling are potentially significant metabolisms in these ultradeep environments. To conclude, this study provides information on microbial ecology of low biomass, carbon-depleted and energy-deprived deep subsurface environment. This information is useful in the prospect of finding life in other planetary bodies.

## 1. Introduction

Currently, Earth is the only observed inhabited planetary object in the Universe. In order to identify feasible extraterrestrial locations for habitability and potentially life, different approaches have been employed: through calculating habitable zones in solar systems or in the Universe [1], defining what is meant with life and habitability [2], and quantifying the probability of origin of life [3]. Although we have been unable to retrieve tangible samples or incontrovertible evidence from possible extraterrestrial life, the search is ongoing. Using analog environments on Earth, we can likewise explore the great capacity of life to proliferate in multiple extremes [4]. Among these analogs, the deep continental subsurface on Earth provides an example for any subsurface crustal environment in a rocky planetary body or icy moon [2,5,6]. Deep subsurface environments on Earth represent characteristics that are expected to be found in the deep subsurface of other planetary bodies [5,6]. These include a large rock-water ratio, carbon deprivation and oligotrophic conditions, extremes in temperature, salinity or radiation, and likely because of all these, a low amount of biomass [5,7].

The deep terrestrial biosphere has been studied in many sites across the globe. The Canadian Shield and the Kalahari Shield in South Africa represent most ancient bedrock composed of sedimentary rocks [8,9], while Fennoscandian Shield bedrock is composed of granites and gneisses [10,11]. Recent study in India investigated the microbial community structure and functions in Late Cretaceous Deccan traps and the underlying granitic Archaean basement rock [12]. Deep-emanating fluids in the Timmins Mine, Canadian Shield represent approximately 1.5 billion year residence times [13]. South African mines have provided suitable study sites for the deep biosphere more than two decades. Interesting finds, such as single-member community of sulfate-reducing “*Candidatus* Desulforudis audaxviator” in fracture fluids [14], or previously uncharacterized nematode species living in 1.3 km depth have been described from deep subsurface of South Africa [15]. In addition, the cosmopolitan deep subsurface inhabitants, Hadesarchaea were first described from South African gold mines [16,17]. Microbial communities described from several sites in the Fennoscandian Shield share many structural similarities with other deep terrestrial subsurface environments. Proteobacteria and Firmicutes are frequently detected and in many cases the most dominant organisms [12,18,19,20,21,22,23,24,25]. Gases of geological origin, such as hydrogen and carbon dioxide, are considered to be the initial driving force of the microbial community [26]. These provide both energy and a carbon source for autotrophic organisms, which turn inorganic carbon into organic carbon to feed the chemoheterotrophic organisms in the deep subsurface [7,26,27]. All of these findings, in addition to resent expansion of the tree of life with multiple deep subsurface organisms [28,29] provide an opportunity to evaluate the adaptation and survival of life in the deep subsurface. The deep mine in Pyhäsalmi and Otaniemi drill holes, which will be the deepest geothermal heat production plant in the world when finished [30], provide a window to ultradeep subsurface environment and a unique opportunity to explore life deep in the Earth’s crust.

The deep subsurface, albeit providing multiple hardships for microbial life, can still however be a more hospitable environment for life than the surface of a planetary object, including for example the pre-ozone early Earth and during intense periods of meteoric bombardment, where ancient microbes could have sought refuge in the crust [31]. Life can reside at these relatively stable depths for long periods of time, increasing the potential to detect life on other planetary bodies or moons [5]. However, this deep residence is unreachable with current technology [32]. For example, European Space Agency (ESA)’s Exomars rover that is designed primarily to detect signs of life on Mars will carry a drill with a capacity to reach only to two-meter depth [33,34]. As such, understanding the metabolic cycles and by-products of these deeply-residing biospheres can reveal what gas biosignatures may escape to the surface for detection by orbiting spacecraft (e.g., ESA ExoMars Trace Gas Orbiter) or telescope technology.

Despite the protection it offers from inhospitable surface conditions, the deep subsurface is a resource-limited environment. It is often oligotrophic with a low flux of nutrients, and a highly reducing and oxidant-limited environment providing only minimal energy gradients [5,7]. Deep subsurface environments are reactant limiting, although lithogenic electron donors, for example H_2_, are often present [35]. The capacities of microbial life of the modern Earth are currently under investigation with DNA and RNA sequencing methods. These metagenomic approaches provide insights into the metabolic processes and evolutionary history of microbes. In addition, these can be used to assess the possible limits of life especially in multiextreme environments [36].

This study investigated the limitations to life in the crystalline deep subsurface and examined the microbial ecosystem and its metabolic potential in multi-extreme conditions in the ultradeep (up to 4.4 km depth) continental bedrock. This study also highlights the importance of safeguarding the potential planetary habitats from terrestrial contamination from Earth. The future missions could be probing for similar kinds of environments with an extremely low cell content, where forward contamination is a major issue to tackle with.

## 2. Materials and Methods

### 2.1. Site Descriptions

Samples were retrieved from two different study sites: (1) experimental drill hole number R-2247 at the Pyhäsalmi Cu-Zn mine (First Quantum Minerals, Ltd., Vancouver, BC, Canada), Finland, from a drill hole reaching the depth of 2.4 km and (2) OTN2 and OTN3 deep drill holes from a depth range of 2.6–4.4 km in the municipal area of Espoo, Finland. Site description for the drill hole in Pyhäsalmi mine has been previously published [37]. Briefly, Pyhäsalmi mine is located nearby Pyhäjärvi town, central Finland (26.042° E, 63.659° N). The exploratory drill hole was drilled in 2012 into tonalitic and metavolcanic rocks and is naturally overflowing but has been plugged since the drilling. The drill hole R-2247 fluids are saline Ca-Na-Cl type that contain 0.093 mM of total organic carbon and 0.066 mM dissolved organic carbon. Dissolved carbon dioxide and methane have previously been detected in the gas phase (0.012 mL·L^−1^ and 4.08 mL·L^−1^, respectively) [37]. The Otaniemi deep drill hole is located in Aalto University campus in Espoo, Southern Finland (24.827° E, 60.188° N). Drill holes OTN2 and OTN3, drilled in 2016–2017 are designed for geothermal heat production by St1 Deep Heat Ltd. A pilot drill hole reaching a depth of 2 km was drilled and explored prior to drilling of the deeper production wells, which provided information about the fracturing and temperature of the bedrock in the vicinity of the OTN2 and OTN3. The drill hole sampled in this study was at the time drilled to the depth of 4.5 km, using the air hammer drilling technique. Estimated in situ temperatures range from 46 to 76 °C in sampling depths of this study [38]. Bedrock in the Otaniemi area comprises of mainly of mica gneiss and migmatitic granite [39].

### 2.2. Sampling

Fluid samples for microbiological analyses were retrieved from Pyhäsalmi R-2247 in 26th of April 2016 by first unplugging and flushing the drill hole for five hours. The flow rate was approximately 40 L·h^−1^. An acid-washed, autoclaved pressure-tight stainless-steel cylinder was fastened to the tap of the drill hole plug, flushed with circa 5 L of fluid and closed after it was filled to the top. Altogether seven parallel 0.5 L samples were retrieved. The cylinders were kept at room temperature (close to ambient temperature of the fluids in the mine, 23 °C), transported to the laboratory and stored 9 days prior to analysis. The valves were flame-sterilized and cylinder valves were gradually opened under N_2_ gas flow in order to allow sample gas pressure to gradually stabilize to normal pressure. Samples were emptied from the cylinders into acid-washed, sterile Schott bottles under N_2_ flushing. Biomass from six samples was immediately collected by filtering the sample through 0.2 μm Sterivex filters (Merck-Millipore, Merck KGaA, Darmstadt, Germany). Three filters with biomass were dedicated for DNA analysis (Pyhäsalmi DNA a–c) and three for RNA analysis (Pyhäsalmi RNA a–c). Filters dedicated for RNA analysis were filled with LifeGuard solution (Mo Bio, QIAGEN Inc., Hilden, Germany) for better preservation of RNA in the samples. All filters were stored in sterile 50 mL Corning tubes at −20 °C prior to further analysis.

Geochemical parameters (dissolved O_2_, pH, electrical conductivity and temperature) were measured in a flow-through cell with portable sensors (WTW) at the beginning of the flushing and immediately after collecting the microbiological samples. In addition, samples were taken for geochemical laboratory analysis after ca. 1 hour of flushing (Geochemistry sample a) and just before the microbiological sampling (Geochemistry sample b). Filtered (<0.45 μm) 100 mL samples were taken for cation analysis and 250 mL and 500 mL unfiltered samples devoted to anion analyses and determination of alkalinity, respectively. Two 100 mL samples were also taken for sulfide analysis prior to microbiological sampling. These were collected in glass bottles (Winkler) and immediately fixed with 2 M NaOH and 1 M zinc acetate. Cation samples were acidified with ultrapure HNO_3_ and all geochemical samples stored at +4 C prior to analysis. Alkalinity was determined by end-point titration to pH 4.5 in the same evening using a digital titrator (Hach, Loveland, CO, USA), and other samples brought to commercial laboratories for cation and anion analysis (Labtium Oy, Espoo, Finland), and sulfide analysis (Ramboll Oy, Vantaa, Finland).

Crushed rock material from Otaniemi drill holes OTN2 and OTN3 was gathered into a plastic sample collection bucket at the airflow output of the drilling apparatus. Samples were collected from the plastic bucket by grabbing a handful of crushed rock material with a UV-sterilized plastic bag and turning it inside out, while avoiding touching the sides of the bucket. Excess air was squeezed out from the bags and samples were frozen at −80 °C for further analysis. Two replicate rock material samples were taken from each sampling depth. Samples were collected from OTN2 on 1st of July and 9th of August, 2016 (Otaniemi 1, 2569 m and Otaniemi 7, 3115 m, respectively) and from OTN3 from 29th of October to 15th of November on five occasions (Otaniemi 2, 3, 4, 5 and 6, depths respectively 4015, 3203, 4375, 4203 and 3617 m). As air was used in the drilling to flush out the crushed rock material from the borehole in Otaniemi, we collected air samples in order to detect possible airborne contaminants of the samples. We filtered 1 m^3^ of air during 35 min using an Impactor FH5^®^ sampler (Markus Klotz GmbH, Bad Liebenzell, Germany). Airborne particles were retained on a gelatin filter paper (Gelatin Filter Disposables, Sartorius Stedim Biotech GmbH, Göttingen, Germany), which were further processed as the other filter samples. Air samples were collected twice, 11th of July and 1st of November 2016.

### 2.3. Sample Preparation for Molecular Biology Analyses

All molecular biology procedures were carried out in a laminar flow cabinet sterilized with UV light and before RNA extraction, surfaces were also wiped with RNase Zap wipes (ThermoFisher Scientific, Waltham, MA, USA). DNA and RNA extraction from Pyhäsalmi mine fluid samples was done with and NucleoSpin Soil (DNA) and NucleoSpin RNA plant (RNA) (Macherey-Nagel, Düren, Germany) kits. First, the Sterivex filter case was cut open with flame-sterilized tools and filter cut out with a sterile scalpel. The filter was cut into small slices and placed to the extraction tube of the kit. In the RNA extraction, the LifeGuard solution left in the Sterivex units was also pipetted to the extraction tube. DNA and RNA were then extracted according to the manufacturer’s instructions. A sterile filter for extraction control was treated as samples. After extraction, DNA yield was measured with Nanodrop spectrophotometer (ThermoFisher Scientific) and DNA was kept at -20°C for further analysis. RNA was translated to cDNA with Sensifast cDNA Synthesis kit (BioLine, London, UK) according to the manufacturer’s instructions, aliquoting each RNA sample into four parallel reactions that were combined after translation.

Two methods were used for DNA extraction from Otaniemi rock samples, either straight extraction with TriPrep kit (Macherey-Nagel) using 1 g of ground rock sample, or washing procedure combined to nucleic acid extraction. The washing procedure was done with mixing 100 g of rock material with 500 mL Na-phosphate buffer (1M, pH 7), shaking (30 min, 200 rpm), letting heavier particles sink down for 1.5 h and decanting the supernatant to cellulose acetate filter (Corning Inc., Corning, NY, USA) in order to collect the biomass by filtration. After filtration, the filters were cut out of the funnels with a sterile scalpel, halved and frozen at −20 °C in test tubes. Nucleic acid extraction from the filters was performed as described for the Pyhäsalmi samples above. Nucleic acid yield was measured with Qubit (ThermoFisher Scientific). Negative extraction controls as well as air controls were included in the analysis and treated as actual samples.

### 2.4. Sequencing of the Microbial Community

The microbial community structure of Pyhäsalmi R-2247 was determined with unidirectional 16S rRNA gene amplicon sequencing of DNA and cDNA with the IonTorrent PGM platform at Biocenter Oulu sequencing center (University of Oulu, Finland).The 16S rRNA gene V3-V4 region amplicon libraries were produced using MyTag mastermix (Bioline, Memphis, TN, USA) and primers 341f and 785r [40] for bacteria and 349f-806r primers for archaea [41] (Table S1). For the fungal ITS1 gene region ITS1 and ITS2 primers were used [42,43]. The amount of template DNA was increased to 4 μL, after first experimenting with 2 μL. The thermal cycle program used to amplify the libraries was as follows: initial annealing 94 °C for 5 min, 45 × 94 °C for 1 min, 50 °C for 30 s, 72 °C for 1 min, and final extension at 72 °C for 10 min. After purification and size selection, amplicons were sequenced using the 316 Chip Kit v2 with Ion PGM Template IA 500 and Ion PGM Hi-Q Sequencing kits (Thermo Fisher Scientific).

The bacterial community structure of the Otaniemi samples was determined with paired-end (2 × 150 bp) 16S rRNA gene amplicon sequencing (V4-V5 region) from DNA retrieved with the washing procedure combined with kit extraction. Sequencing was performed with Illumina MiSeq platform in Marine Biology Laboratory (Woods Hole, MA, USA) according to their online protocol (https://vamps2.mbl.edu/resources/primers, accessed 10.12.2019). Briefly, DNA concentration of the samples was first determined with PicoGreen assay (ThermoFisher Scientific) then samples were concentrated with speedvac. PCR amplification was done with 35 cycles instead of the default 30. Samples were cleaned using AMPure beads (Beckman Coulter Life Sciences, Indianapolis, IN, USA) according to manufacturer’s instructions and quantified prior to sequencing.

In addition, two Pyhäsalmi R-2247 samples (Pyhäsalmi DNA b and c) were sent to the Marine Biology Laboratory (MA, USA) for paired-end metagenomic sequencing with the Illumina MiSeq, resulting to ~275 nt long products, as part of the Deep Carbon Observatory’s Community of Deep Life sequencing effort. Due to low amount of DNA, multiple displacement analysis was performed prior to preparation of the metagenomic libraries. Nugen Ovation UltraLow DR Kit was used to prepare the metagenomic libraries otherwise according to the manufacturer’s instructions, but with increased number of amplification cycles from the suggested 18 to 22.

### 2.5. Quantification of Taxonomic and Functional Marker Genes

The quantities of different taxonomic and functional groups of microbes were determined with quantitative PCR. Bacterial and archaeal numbers were determined with amplification of 16S rRNA gene with domain-specific primers [44,45,46,47]. Sulfate and nitrate reducing microorganisms in addition to methanogens were quantified using functional gene copy numbers. Functional genes amplified with qPCR were *dsr*B, *nar*G and *mcr*A, respectively. Primers and standards for each assay are described in Supplementary Material, Table S1 [48,49,50,51,52]. Roche LightCycler SYBR Mastermix with 1 μL of bovine serum albumin, 10 μM of forward and reverse primer and 1 μL of nucleic acid template was used for PCR mastermix. Quantitation was done with LightCycler 420 (Roche Molecular Diagnostics, Pleasanton, CA, USA) using the following protocols: 5 min at 95 °C, 40 cycles of 95 °C for 30 s, 60 °C for 30 s and 72 °C for 15 s, and final extension of 72 °C for 3 min. Sample fluorescence was measured at the end of each elongation phase. Melting curve analysis comprised of 15 s denaturation at 95 °C, 1 min annealing phase at 55 °C (archaeal 16S rRNA and *mcr*A assays) and or 65 °C (bacterial 16S rRNA, *nar*G and *dsr*B assays) and continuous measuring and melting step with temperature rising 0.11 °C per s to 95 °C.

### 2.6. Sequence Data Analyses

For the 16S rRNA gene profiling, the data were processed using MOTHUR v.1.39.5 [53]. The Otaniemi data was analyzed using the MOTHUR MiSeq SOP (https://www.mothur.org/wiki/MiSeq_SOP), while Pyhäsalmi samples (sequenced with IonTorrent) were analysed as described in Purkamo et al. (2017) [54]. Otaniemi sequence data was trimmed with default parameters, while Pyhäsalmi bacterial sequences were trimmed with the following parameters: maxambig = 0, maxhomop = 8, bdiffs = 0, pdiffs = 2, qaverage = 25, minlength = 200, while parameters for archaea were maxambig = 0, maxhomop = 8, bdiffs = 0, pdiffs = 0, qaverage = 25, minlength = 200 and fungi maxambig = 0, maxhomop = 8, bdiffs = 0, pdiffs = 0, qaverage = 15, minlength = 120. Bacterial and archaeal OTUs were clustered using 97% similarity. Taxonomic classification was done for all 16S rRNA gene sequences using the SILVA alignment v. 132. Fungal ITS reads were Blast-searched against UNITE database v.8.0 [55]. The OTUs detected in DNA extraction control and PCR-negative control sample in Pyhäsalmi’s case were removed from the final sequence dataset using get.groups, list.seqs and remove.seqs commands.

Metagenomic reads from Pyhäsalmi were run through EBI Metagenomics pipeline v.4.0 using default settings [56]. Reads were quality checked and trimmed for low-quality regions and adapter sequences using Trimmomatic v. 0.35 [57]. Gene-coding regions were searched with FragGeneScan v. 1.20, InterProScan v. 5.25-64.0 and Prodigal v. 2.6.3. Sequence and structural similarities to noncoding RNA sequences were searched with Infernal v. 1.1.2 and rRNA sequences classified with MAPseq v. 1.2 using Silva 128 reference database. Metagenomic reads were also trimmed in Galaxy web platform (www.usegalaxy.org) [58] using Trimmomatic v.0.38.0 with ILLUMINACLIP, SLIDINGWINDOW and MINLEN trimming (parameters used: nr of bases to average across = 4, average quality = 20, minimum length of reads to be kept = 50) [57]. Taxonomic labels were assigned to trimmed reads with Kraken v. 1.3.0 [59] using database for bacteria, Kraken data translation to full NCBI taxonomy (v. 2015-15-10), and further visualized with a Krona chart. Metagenomic sequences were annotated with KAAS protein annotation tool using GHOSTX search with single-directional best-hit method, and KEGG’s GhostKOALA annotation program generating KEGG Orthology assignments and reconstructing KEGG modules and pathways [60,61]. Reads were assembled to contigs with Megahit v. 1.1.2 [62] with default parameters.

Microbial community functionality in Pyhäsalmi and Otaniemi samples was also predicted with FAPROTAX [63]. Taxonomy of the microbial communities was compared against the FAPROTAX database using the Microbiome Helper [63,64]. Relative functionality abundances and assigned functions for each sample were further visualized in R (R Core Team, 2018) with package ggpubr [65].

### 2.7. Diversity Indices

Shannon (H′) and Simpson diversity indices in addition to species richness (Chao1) and coverage (ACE) estimates were calculated using the MOTHUR-generated .biom-file in R with Phyloseq package version 1.20.0 [66].

### 2.8. Data Deposition

MBL-generated sequences from the Otaniemi 16S rRNA gene study are freely available at the VAMPS database (http://vamps.mbl.edu) under the project code DCO_PUR_Bv4v5. The sequences are also available in ENA (accession numbers ERS4143321-24. Pyhäsalmi 16S rRNA and ITS sequences are deposited to EBI under accession numbers ERS3941186-93 (bacteria) ERS3941385-90 (archaea) and ERS3941391-94 (fungal ITS). Metagenomic sequences are deposited in EBI’s MGnify platform under study MGYS00001946 (study number PRJEB22782, sample accession numbers ERS1940559 and ERS1940560).

## 3. Results

### 3.1. Geochemistry

Geochemical data collected in this study is presented in Table 1, and field measurements in Table 2. Geochemistry has been previously characterized in Miettinen et al. (2015) [37], and no significant changes in the composition of the fluid was observed. Ca and Na are the most dominant cations, and Cl is the most abundant anion. Total dissolved solids are up to 81 g L^−1^, demonstrating the high salinity of the fluids (Table 1). While other parameters measured in the field were constant compared to previous report, a slight rise in pH could be observed (Table 2).

### 3.2. Low Biomass Environment

**Figure 1:**
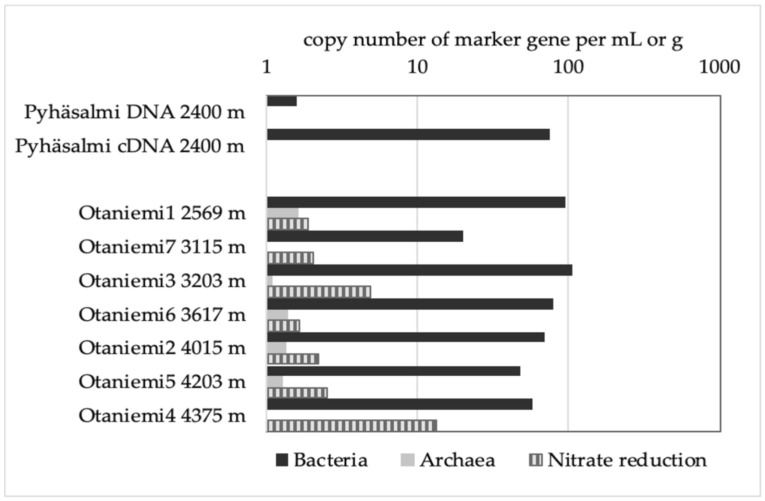
Copy numbers of bacterial, archaeal phylogenetic marker genes (16S rRNA), and nitrate reduction marker gene (*nar*G) in Pyhäsalmi DNA and cDNA samples (average of three parallel samples each) and Otaniemi DNA samples.

Both our sampling sites represent ultralow biomass environments. Bacteria were universally more common in the studied ultradeep biospheres compared to other microorganisms according to cell number estimates measured with quantitative PCR. The copy numbers (used as a proxy for the number of cells) showed 2–76 copies of bacterial cells m L^−1^ in Pyhäsalmi samples (Figure 1). Archaeal 16S rRNA gene copies were below the detection limit (40 copies/ml or g of sample) in Pyhäsalmi, and could be only estimated by extrapolating from the standard curve. Otaniemi crushed rock samples contained 20–105 copies of bacterial and 1–2 archaeal 16S rRNA genes per gram. Nitrate reduction (*nar*G) marker gene copies were detected from Otaniemi samples, copy numbers ranging from 2–13 per mL.

The higher relative abundance of bacteria vs. archaea and fungi was also demonstrated with phylogenetic marker gene amplicon sequencing. Pyhäsalmi samples resulted in a total 24 910 bacterial reads (number of sequences in samples ranging from 1624 to 5864), while archaeal 16S rRNA gene sequencing resulted to only 14 and fungal ITS gene region sequencing to 11 reads.

Extraction control and PCR negative control resulted in 637 and 1476 bacterial reads, respectively. Amplicon sequencing of the bacterial 16S rRNA gene in the Otaniemi samples resulted in a total of 270k reads. Of this, the shallowest depth (3203 m) contained 62% (167,290 reads), sample from 4203 m 16% (42,413 reads) and deepest depth at 4375 m 12% (28,779) of the total reads. Approximately 11% of reads were obtained from extraction control. Sequencing was not successful from control air samples and PCR negative control. Although extraction control also resulted in a number of reads, the OTUs those represented were removed from the data prior to diversity analyses and functional prediction with FAPROTAX. Pyhäsalmi metagenomes resulted in libraries of 221 Mb and 130 Mb.

### 3.3. Microbial Community Composition

**Figure 2:**
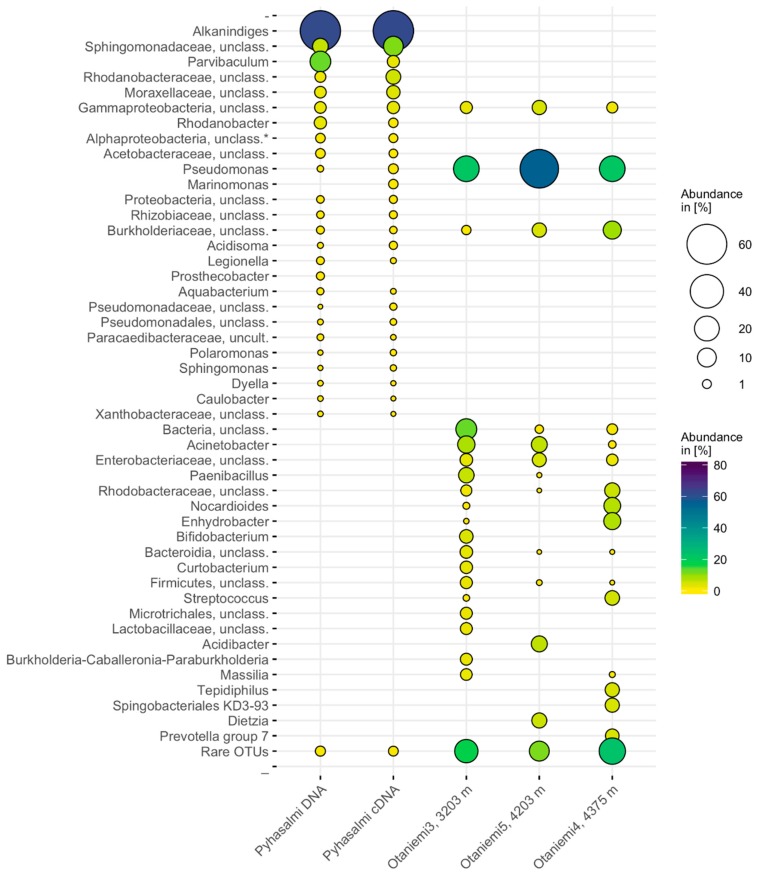
Bacterial community structure based on 16S rRNA gene amplicon sequencing at Pyhäsalmi and Otaniemi deep drill holes. Results from three replicate samples of Pyhäsalmi have been combined and average relative abundance is shown. OTUs detected from controls are filtered out from the result, except for one unclassified Alphaproteobacterial OTU present in Pyhäsalmi samples (marked with an asterisk, *). Rare OTUs represent those OTUs that were present less than on average 1% or 0.1% relative abundance in the samples from Otaniemi and Pyhäsalmi, respectively.

While the samples contained extremely low biomass, we retrieved more than 200 different 16S rRNA OTUs with the community sequencing effort from both study sites. OTUs representing on average less than 1% of the total bacterial community in each sample formed approximately 10–20% of the bacterial communities, thus representing the rare biosphere. Altogether 286 bacterial OTUs were detected from Pyhäsalmi mine samples originating from 2.4 km deep drill hole. According to phylogenetic marker gene amplicon sequencing, the majority of the microbial community of this drill hole in Pyhäsalmi comprised of Proteobacteria, mainly Alpha- and Gammaproteobacteria (on average 96%) (Figure 2).

The Shannon diversity estimate H′ ranged from 2.3 to 2.4 between the Pyhäsalmi samples (Table 3). A phylotype closely affiliating with *Alkanindiges* (Gammaproteobacteria) was the most dominant detected in both the DNA and RNA fraction, with a relative abundance of 56–64% of the bacterial community. In the DNA-derived bacterial community, the second most common OTU affiliated with *Parvibaculum* (Alphaproteobacteria), while in the RNA fraction, an OTU affiliating with *Sphingobacteriaceae* (Bacteroidetes) had higher relative abundance. On average, according to the Chao1 estimate, 53% of the richness of the community detected from the DNA fraction and 57% of the RNA fraction was captured (Table 3).

The abundance-based coverage estimate (ACE) showed that on average 52% and 49% of the bacterial richness was captured in sequencing the DNA and RNA fractions, respectively (Table 3). The DNA and RNA communities shared 59 OTUs (34.5% of all the OTUs). Of the negative DNA extraction control, 90% of the sequences affiliated with betaproteobacterial *Ralstonia*, which was not detected in any of the actual samples (Supplementary Material, Table S2). In the PCR negative control, the most common OTUs affiliated with different Cyanobacteria (52%). These were rare, on average 0.02% of the sequences from all the subsurface samples. However, 5% of the sequences in the PCR negative control sample affiliated with an alphaproteobacterial phylotype that was also present in the samples with 1–2% relative abundance. The only archaeal sequences detected with 16S rRNA gene sequencing affiliated with *Methanobrevibacter (Methanobacteria)* and thaumarchaeotal “*Candidatus* Nitrosopumilus”. Fungal ITS sequences affiliated with ascomycotal *Cladosporium* and *Orbilia*, and basidiomycotal *Vuilleminia*, *Apiotrichum* and *Trichosporon* (98–100% identity score from BLAST). Some sequences could only be identified to kingdom level (i.e., to Fungi). With a very few sequences per each sample, ecological indices were not calculated for archaeal and fungal sequences.

Sequencing was successful from three different depths of Otaniemi OTN3 drill hole samples (3, 4 and 5). Bacterial communities from these depths had distinct compositions (Figure 2). Altogether 203 OTUs were observed from the data of which DNA extraction control was filtered out. Of these, the samples shared 27 OTUs. The bacterial community in Otaniemi depth at depth of 3203 m comprised mainly of Gammaproteobacteria (37%), Firmicutes (15%), Actinobacteria (13%), unclassified Bacteria (13%) and Bacteroidetes (10%). This depth had also the highest diversity index, Shannon *H*′ = 3.9 (Table 3). Gammaproteobacteria formed the majority of the bacterial community at 4203 m depth (78%). Betaproteobacteria (*Burkholderiaceae*–affiliating OTU, 5%) and Actinobacteria had the next highest relative abundance (mostly *Dietzia*, 5% relative abundance). The Shannon diversity index *H*′ was 3.4. The major groups in the 4375 m sample were Gammaproteobacteria, 53%, Actinobacteria 15% (of which a *Nocardioides*-affiliating OTU representing 7% relative abundance), and Bacteroidetes (10%). This sample had the *H*′ index of 2.5, which was the lowest of all studied Otaniemi samples. Most common gammaproteobacterial OTUs affiliated with *Pseudomonas*, *Acinetobacter*, *Enhydrobacter* and unclassified *Enterobacteriaceae*. The second-most common OTU in the Otaniemi bacterial communities could not be classified further than to phylum level (unclassified Bacteria). Off all the control samples taken, only the DNA extraction control yielded sequences, of which most were affiliated with Gammaproteobacteria. Of these, *Pseudomonas*-affiliating OTUs were also detected in the samples, but for example *Solirubrobacter*, *Rheinheimera*, *Afipia* and unclassified *Burkholderiaceae*–affiliating sequences were only detected in large quantities in the DNA extraction control (Supplementary Material, Table S2).

The metagenomic data of the microbial community structure in Pyhäsalmi supports loosely the amplicon sequencing results. From the two combined metagenomes, most of the reads were assigned to bacteria, 97% of the total of 63,255 sequences (Supplementary Material, Figure S1). According to Kraken, the majority of the bacterial community at 2.4 km depth at Pyhäsalmi comprised of Proteobacteria (on average 39%). Actinobacteria (38%) and Firmicutes (15% were also present in the combined metagenome. Alphaproteobacterial *Rhizobiales*, betaproteobacterial *Burkholderiales* and gammaproteobacterial *Pseudomonadales* represented the most abundant orders. Actinobacteria and Firmicutes composed of several presumably contaminant taxa, namely *Propionibacterium*, *Streptococcus* and *Staphylococcus*. Archaeal sequences formed 1% of the total community with *Methanosarcinales* the most abundant order. Thaumarchaeota represented 1% of the archaeal community.

### 3.4. Microbial Functionality

Microbial functionality was tested with marker gene assays using quantitative PCR. Out of the tested marker genes, only *nar*G gene copies were detected. These nitrate reduction marker genes were successfully quantified from 3203 m and 4375 m at Otaniemi (on average five and 13 copies of *nar*G per g of sample, respectively) (Figure 1). As the detection limit of this assay is 10 copies per g of sample, we could only extrapolate numbers from the standard curve for the 3203 m sample. Sulfate reduction and methanogenesis marker gene copies were not detected from either site.

**Figure 3:**
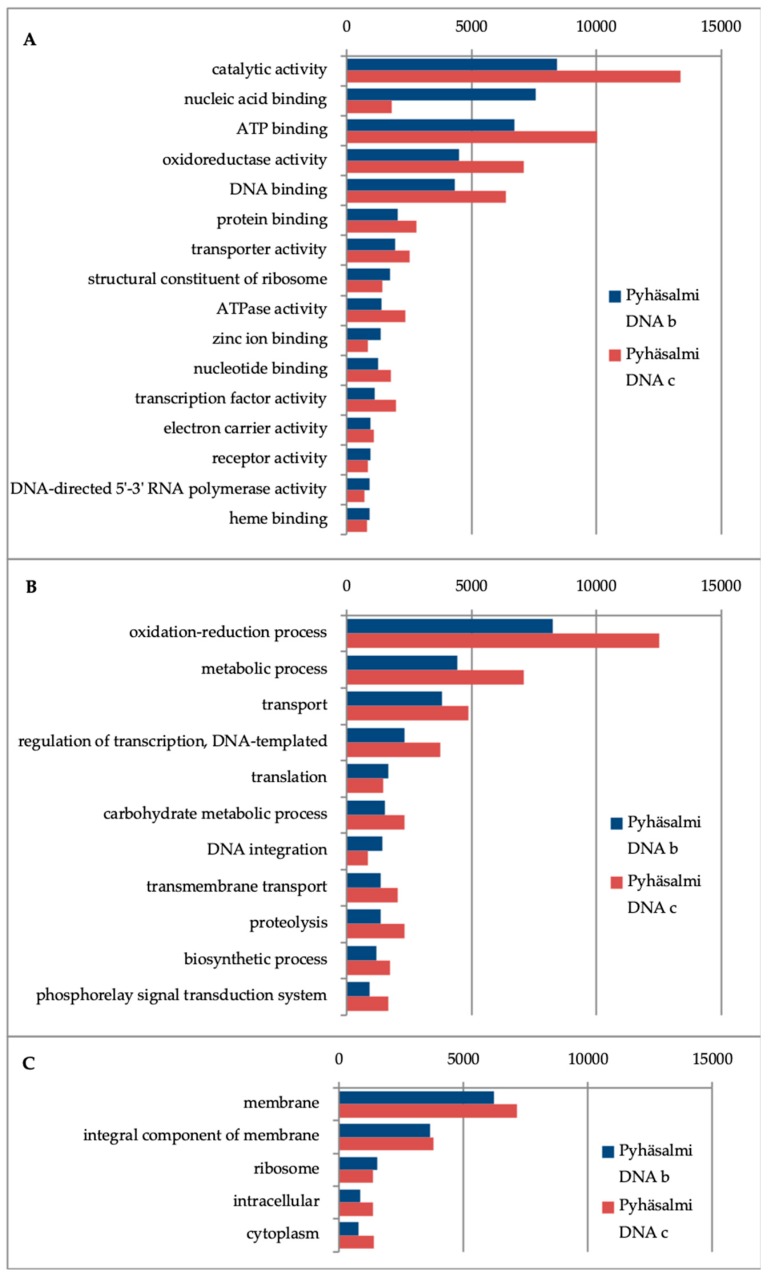
Gene ontologies discovered from two replicate Pyhäsalmi metagenomes related to (**A**) molecular function, (**B**) biological process, and (**C**) cellular component-categories.

Looking into the functions in Pyhäsalmi metagenomes, the most frequent gene ontology annotations in biological process category were metabolic and biosynthetic process, nitrogen compound and small molecule metabolic process, and processes involved in transport (Figure 3). In the molecular function category ion, nucleic acid and nucleotide binding, oxidoreductase and catalase activities were the most prevalent categories. Gene ontologies that were most abundant in the cellular component category were membrane and intrinsic to membrane-categories.

In order to understand more deeply the ecosystem functionality of the microbial community, we used KEGG’s GhostKOALA annotation for the Pyhäsalmi metagenomes. Reconstructed KEGG modules revealed the complete module of the reductive pentose phosphate pathway (Supplementary Material, Table S3). Complete modules for gluconeogenesis, pyruvate oxidation, pentose phosphate pathway, glyoxylate cycle, and several amino acid biosynthesis modules were also detected. In the environmental information processing category, nitrate/nitrite transport system in addition to for example phospholipid, ribose, peptide/nickel and ABC transport systems were complete. Dissimilatory nitrate reduction pathway was fully reconstructed using KAAS (Supplementary Material, Figure S2).

The Megahit assembly resulted in 3502 contigs with a total of 1.4 Mbp. The average contig length was 408 bp, ranging from minimum of 200 bp to maximum of 9 kbp. The N50 was 398 bp. With this amount of contigs and short average contig length, no further analysis was attempted for the metagenomic data.

The functional profiles from FAPROTAX analysis had 370 and 57 assignments affiliating to at least one group in Otaniemi and Pyhäsalmi samples, respectively. Otaniemi samples hosted 28 different functional groups, whereas Pyhäsalmi samples hosted 32 different functional groups. Some of the detected microbial community members in Otaniemi and Pyhäsalmi remained unclassified and represented uncultured species, whereas the FAPROTAX database relies on the characterized strains. In detail, 89% of the Otaniemi OTUs and 80% of the Pyhäsalmi OTUs were left without functional assignment based on FAPROTAX database.

**Figure 4:**
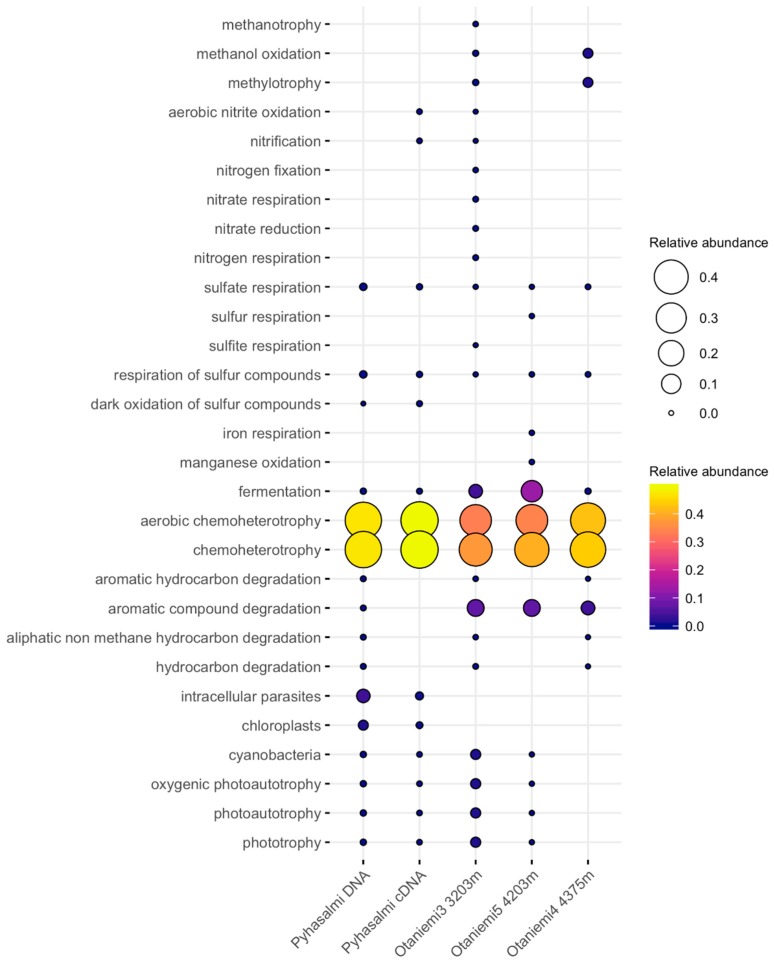
Relative abundance of different cellular functions (Y axis) based on the FAPROTAX database in Pyhäsalmi and Otaniemi samples. Both the size of the balloon and the color indicate the relative abundance of taxonomy-based functional capacities of the microbial community.

Functional FAPROTAX predictions indicated that chemoheterotrophy represented a major driving force in deep biosphere metabolism in both Otaniemi and Pyhäsalmi mine (Figure 4) (Supplementary data, Data file S1). OTUs grouped with chemoheterotrophic lifestyle belong to Alphaproteobacteria, Gammaproteobacteria, Actinobacteria and Firmicutes. Same OTUs appeared also in the aerobic chemoheterotrophy-category, likely due to the nature of the bacterial species (facultative anaerobes) these OTUs were assigned. In Pyhäsalmi, the metabolic profiling indicates possible sulfate and sulfur compound respiration. The metabolic profiles of Otaniemi microbial communities show potential for fermentation, methylotrophy and aromatic carbon compound degradation. The functional fermentation group was detected based on orders such as *Clostridiales*, *Bacteroidales* and *Pseudomonadales*. Methanol oxidation and methylotrophy are potential metabolisms at Otaniemi at depth of 4375 m. Minor levels of sulfate metabolism (0.01–0.3% of detected functional groups), were detected, and sulfate and sulfur compound respiration in Otaniemi and Pyhäsalmi could be linked to *Desulfobacterales* (Supplementary data, Data file S1). There is potential for more complex metabolic pathways in Otaniemi microbial communities, such as degradation of aromatic and hydrocarbon compounds.

## 4. Discussion

For more than two decades NASA’s Mars exploration program has been themed “Follow the Water”. Traditionally the habitability of the planet has been defined with the possibility of liquid water existing on the surface of the object. Today we know that life survives and thrives even in the deepest realms of the oceans, in the subsurface sediments and even in cracks and fractures of deep, crystalline bedrock [21,22,67,68,69]. This study, as well as other deep subsurface investigations show, that the deep subsurface habitats usually retain very low cell numbers [70,71,72,73]. However, the deep biosphere biomass consists of approximately 15% of the total biomass of the Earth [74] and therefore could play a significant role in dynamics of elemental cycling on all inhabited planetary objects.

### 4.1. Habitats Hosting Low Biomass

Multi-extreme surface habitats on planetary bodies are considered inhospitable [75]. However, subsurface could provide a more suitable environment for microbial life, although if analogous to Earth, low biomass can impede the detection of life in these environments. Detection of functional molecules, such as DNA and RNA would be a powerful indicator of life on other planetary bodies, and could be regarded as the smoking-gun evidence [76], assuming that extraterrestrial life is DNA- or RNA-based. Quantitative PCR methods are very sensitive and work well with very low amount of DNA [77]. With quantitative PCR, there would be possibility to retrieve information about the volume and functionality of life, assuming that life on other planetary objects would not be excessively different from life on Earth. In the present study, we were able to demonstrate the feasibility of qPCR in determining life in ultralow biomass habitat, with detection of fewer than ten to some hundreds of copies of 16S rRNA gene and transcript fragments from the extracted DNA and RNA. Such an approach demonstrates a targeted need for sample return missions.

### 4.2. Microbial Community Structure

The microbial communities detected from both our study sites had a few dominating OTUs. In Pyhäsalmi, more than half of the 16S rRNA genes and transcripts sequenced affiliated with *Alkanindiges*. The type strain of this bacterium, *Alkanindiges illinoisensis*, isolated from oilfield soil is using long-chain linear and branched hydrocarbons and only grows weakly on acetic acid [78]. Interestingly, *Alkanindiges* 16S rRNA gene sequence is 99.1% similar with unclassified bacterium clone from deep groundwater in Maqarin, Jordan, therefore, this was not the first time for this type of bacterium to be detected from the deep biosphere [79]. No long-chain hydrocarbons have been reported from borehole in Maqarin, but there are small amounts of iso-butane, N-butane and N-pentane reported from Pyhäsalmi R-2247 drill hole [37]. Thus, it is possible that this bacterium uses those as growth substrates in Pyhäsalmi bedrock.

*Pseudomonas* was the most common phylotype in Otaniemi samples. Bacteria related to *Pseudomonas* have been detected in many deep biosphere studies, and it has been suggested to form the core microbiome in deep subsurface [80,81,82]. Although there is speculation about *Pseudomonas* being contaminants in deep subsurface sequence datasets, there are cultivation studies where these microbes have been isolated from deep fluids [83,84]. Other *Pseudomonadales* affiliating bacteria (*Acinetobacter*, *Enhydrobacter*) were also present in relatively high abundance in Otaniemi. *Pseudomonadales* is a heterogeneous order of Proteobacteria that are ubiquitous in many ecosystems including aquatic and soil environments and have significant ecological importance. They can be regarded as “weeds” of the bacterial kingdom, as growth can occur in various different habitats with wide temperature and pH range. As pseudomonads are metabolically versatile chemoorganotrophs, their carbon sources can vary from amino acids to aromatic compounds. Pseudomonads are mainly aerobic organisms, while some are denitrifiers [85,86,87].

The apparent lack of chemoautotrophs in our samples is surprising, as these are usually the main primary producers in the deep biosphere [26]. However, chemoheterotrophic organisms are flexible in their metabolism and therefore might gain a competitive edge against the chemoautotrophs [22,88]. In many deep subsurface environments, autotrophs form only a minor proportion of the total community compared to heterotrophs [69,89] so we may have missed these because of the low biomass in the first place. In addition, recent studies have shown that some ultra-small microbes in the deep biosphere will pass the filters used in this study, therefore introducing bias to the microbial community structure analysis as well as functional profiling of the community [24,90,91]. Some bacteria also decrease in cell size under oligotrophic conditions similar to deep crystalline bedrock fluids [91], and may be lost during the biomass collection step.

The rare biosphere, i.e., OTUs comprising less than 1% of the total microbial community, was present in significant extent in both study sites. The rare biosphere represents an important gene pool and may in fact play a disproportionately large role in biogeochemical cycling [92,93]. Interestingly, we could also detect some archaea that usually represent a minor part of the total community in deep subsurface environments, and some fungal signals as well. Fungi in the deep subsurface are not particularly well characterized. Only recently studies have highlighted their existence in the deep biosphere, and their ecological role is still rather unclear [94,95].

### 4.3. Metabolic Capacities of Microbial Communities

For future astrobiology or sample return missions to Mars or icy moons, we need to define the feasible microbial functional capacities within the subsurface. It is commonly thought that chemolithoautotrophic organisms are the likely organisms that would be best adapted to conditions in Mars [96,97]. The evolutionary emergence of chemolithoautotrophs coincides with the timeframe when conditions on Mars were favorable for life [98]. Chemolithoautotrophs use inorganic carbon (CO_2_) for building biomass and generating energy. In this study, we detected signals of microbial life that uses organic carbon and a variety of different energy metabolisms. For example, the most abundant organisms in the microbial communities used small organic molecules in both ultradeep subsurface sites in this study. The predicted functionality shows that chemoheterotrophy is a common feature of these microbial communities. As trace organics have been detected on Mars, and in plumes ejecting from Enceladus [99,100,101], chemoorganotrophs should not be neglected in future life detection missions. Even though autotrophs are the primary producers of ecosystems on Earth, the number of autotrophs supporting the total microbial community is sometimes much lower in the deep subsurface compared to heterotrophs [23,89]. Therefore, it might be more reasonable to aim the detection of life towards chemoorganotrophs.

However, potential for autotrophy was demonstrated in Pyhäsalmi metagenomes. Complete reconstructed reductive pentose phosphate pathway from KEGG shows that this important mechanism of autotrophic CO_2_ fixation in nature could be functional in ultradeep crystalline bedrock [102].

Nitrate has been detected in mudstone deposits at Gale Crater on Mars and could provide a nitrogen source, instead of Mars atmospheric nitrogen that is much lower (2.6%) compared to Earth (78%) [103,104]. Although nitrate concentration in Pyhäsalmi is below the detection limit of <0.2 mg L^−1^, we found multiple indications on nitrate metabolism playing a role in the ultradeep, oligotrophic subsurface. From Pyhäsalmi metagenomic data, dissimilatory nitrate reduction pathway could be reconstructed, and a complete nitrate/nitrite transport system mapped. One of the archaeal OTUs detected from Pyhäsalmi samples affiliated with “*Candidatus* Nitrosopumilus” that is an autotrophic ammonia-oxidizing thaumarchaeon [105,106]. We could also detect marker gene copies of nitrate reductase *nar*G, which is functioning in the first nitrate reducing step of the dissimilatory nitrate reduction pathway. Putative functions related to nitrogen metabolism from FAPROTAX were predicted as well. Higher nitrate levels were regarded as a sign of habitability in a Mars analog environment and were suggested as a useful guide for finding life on Mars [107]. This idea is reinforced by the detection of nitrate cycling potential in another type of analog environment in our study. Likewise, of other predicted functions with FAPROTAX, sulfur and sulfate respiration and oxygen-dependent methylotrophy could be accomplished in Martian subsurface conditions [97,108]. However, as FAPROTAX analysis demonstrated, only a relatively small percentage (11–20% in this study) of the total diversity in ultradeep bedrock can be assigned to a cultured microbial species, and therefore the metabolic potential of the deep biosphere remains elusive.

When analyzing microbial communities and their metabolic potential in substrate-limited and oligotrophic environments, one must take into consideration that metabolic pathways may be truncated. Microbes gain energy faster by partial oxidation of carbon compounds, producing intermediate metabolites that, with enough biodiversity, act as food and energy sources to other members of the community [109]. Substrates are recycled in a community so effectively that accumulation of end-products does not occur significantly [110], therefore complicating the observation of these possible biosignatures even further in the search for life in the Solar System.

### 4.4. Considerations on Contamination

Contamination is a pressing issue in all studies of low biomass environments. The cell numbers are extremely low, and consequently the risk of contamination from different sources during the sampling and laboratory procedures is high. Although aiming to retrieve samples with aseptic techniques, using precaution in laboratory work and using stringent quality control with sequence analysis, there are still sources of contamination that cannot be ruled out in deep biosphere studies. Deep subsurface studies are often taking advantage of predrilled holes in mines (in this study Pyhäsalmi) or other industrial drilling (Otaniemi) when microbiological sampling has not been considered and typically not suited to the addition of an equipment sterilization step before the drilling. Therefore, implementing controls into each step of the study: sample collection, nucleic acid extraction, PCR, reverse transcription of RNA and sequencing, is fundamental [83].

We followed the moderately stringent contaminant removal methodology suggested by Sheik et al., but our sequencing dataset still had several OTUs that could be considered contaminants (e.g., *Pseudomonas*, *Acinetobacter*, *Sphingomonas*, *Burkholderia*, *Streptococcus*, *Lactobacillus*, *Dietzia*) [83,111]. Most of these are shown to originate from nucleic acid extraction kits, which would be likely used in extracting nucleic acids from samples in sample return missions. However, there are ongoing technology development for isolation and sequencing of nucleic acids in situ on other planetary objects [112,113]. These methodologies would also be able to identify forward contamination that is a concern whenever landing spacecraft to Mars [96]. Nonetheless, similar precautions and quality control in sampling, sample processing and data analysis should be followed whether we are working with low biomass and analog environments on Earth, meteorites or actual extraterrestrial deep subsurface samples.

## 5. Conclusions

In this study we detected diverse bacterial communities in two different deep terrestrial subsurface locations in the Fennoscandian Shield. Archaea and fungi were detected in very low numbers compared to the bacteria. Gammaproteobacterial *Alkanindiges* OTU was dominating in fluids retrieved from 2.4 km depth, while *Pseudomonas*-related OTUs were common in crushed rock samples retrieved from even deeper, up to 4.4. km depth. Many detected OTUs affiliated with bacteria known for chemoheterotrophic metabolism and/or participation to nitrogen cycling. Metagenomic data also indicated potential for nitrate reduction. In conclusion, this study describes the microbial community in low biomass, carbon-depleted and energy-deprived deep subsurface environment. The information retrieved can be useful for future space missions in the quest of searching life signs in other planetary objects. Missions can be aimed to detect heterotrophic life in subsurface, and if successful, comparison of the Martian life to the deep biosphere found on Earth can be done.

## Figures and Tables

**Table 1:** Groundwater composition in the Pyhäsalmi mine drill hole R-2247. Sample a was taken after one hour of flushing the drill hole, and sample b immediately before the microbiological sampling.

Geohemistry Variable	units	Sample a	Sample b
Alkalinity	mmol/L	0.16	0.17
Total dissolved solids	g/L	77	81
Cations:			
Al	µg/L	5.76	6.50
As	µg/L	0.94	0.61
B	µg/L	728	678
Ba	µg/L	902	928
Be	µg/L	0.16	0.12
Co	µg/L	0.41	0.40
Cr	µg/L	6.46	6.28
Cu	µg/L	0.62	0.58
K	mg/L	57.4	56.1
Mn	µg/L	15.2	14.1
Mo	µg/L	1.89	1.89
Ni	µg/L	4.77	26.6
P	µg/L	47.0	60.2
Pb	µg/L	0.42	0.20
Rb	µg/L	283	282
Se	µg/L	0.41	0.73
V	µg/L	2.39	2.28
Zn	µg/L	2.47	1.63
Ca	mg/L	20500	21500
Fe	mg/L	0.14	0.21
Li	mg/L	0.337	0.322
Mg	mg/L	3.22	3.44
Na	mg/L	7190	7420
S	mg/L	88.7	87.9
Si	mg/L	2.60	2.99
Sr	mg/L	254	266
Anions:			
I	mg/L	7.15	6.98
Br	mg/L	510	510
Cl	mg/L	48000	50000
SO4	mg/L	340	320
NO3	mg/L	<0.2	<0.2
Sulfide	mg/L	-	1.2

**Table 2:** Geochemical on-line measurements of the fluid in the Pyhäsalmi mine drill hole R-2247 before and after the microbiological sampling, and comparison to the previous report [37].

Measurement	units	2016 Before	2016 After	2013	2014
EC	mS/cm	102.6	102.9	103.3 ^1^	102.0 ^1^
pH		9.2	9.3	8.6 ^1^	8.7 ^1^
T	°C	23.6	23.3	23.4	24.0
O_2_	mg/L	0.05	0.04	0.03	NM ^2^

^1^ Adapted from Miettinen et al. [37]; ^2^ NM: not measured.

**Table 3:** Alpha-diversity indices calculated from sequences retrieved from three different depths in Otaniemi and DNA and cDNA samples from three combined replicate samples from Pyhäsalmi.

Sample ID	Depth (m)	Observed	Chao1	ACE	Shannon	Inv. Simpson	Obs./Chao1	Obs./ACE
Pyhäsalmi DNA	2400	115	218	223	2.3	2.5	53%	52%
Pyhäsalmi cDNA	2400	115	202	234	2.4	2.5	57%	49%
Otaniemi3	3203	1032	7440	20,614	3.7	15.9	14%	5%
Otaniemi5	4203	714	5173	14,199	3.4	14.0	14%	5%
Otaniemi4	4375	787	4718	8944	2.5	5.3	17%	9%

## Supplementary Materials

The following are available online at https://www.mdpi.com/2075-1729/10/1/2/s1: Table S1: Details on the primers used in this study. Table S2: Raw counts of OTUs in Pyhäsalmi and Otaniemi samples. Table S3: GhostKOALA module reconstructs from Pyhäsalmi metagenome. Figure S1: Krona chart of the microbial community from Pyhäsalmi metagenome based on Kraken analysis, Figure S2: KAAS produced Kegg pathway for nitrate reduction, Data file S1: FAPROTAX reports.

## References

[1] Rekola R.T.F. (2009). Life and habitable zones in the Universe. Planet. Space Sci..

[2] Cockell C.S., Bush T., Bryce C., Direito S., Fox-Powell M., Harrison J.P., Lammer H., Landenmark H., Martin-Torres J., Nicholson N. (2016). Habitability: A Review. Astrobiology.

[3] Scharf C., Cronin L. (2016). Quantifying the origins of life on a planetary scale. Proc. Natl. Acad. Sci. USA.

[4] Harrison J.P., Gheeraert N., Tsigelnitskiy D., Cockell C.S. (2013). The limits for life under multiple extremes. Trends Microbiol..

[5] Jones R.M., Goordial J.M., Orcutt B.N. (2018). Low energy subsurface environments as extraterrestrial analogs. Front. Microbiol..

[6] Preston L.J., Dartnell L.R. (2014). Planetary habitability: Lessons learned from terrestrial analogues. Int. J. Astrobiol..

[7] Escudero C., Oggerin M., Amils R. (2018). The deep continental subsurface: The dark biosphere. Int. Microbiol..

[8] Stotler R.L., Frape S.K., Ruskeeniemi T., Ahonen L., Onstott T.C., Hobbs M.Y. (2009). Hydrogeochemistry of groundwaters in and below the base of thick permafrost at Lupin, Nunavut, Canada. J. Hydrol..

[9] Onstott T.C., Lin L.-H.H., Davidson M., Mislowack B., Borcsik M., Hall J., Slater G., Ward J., Lollar B.S., Lippmann-Pipke J. (2006). The origin and age of biogeochemical trends in deep fracture water of the Witwatersrand Basin, South Africa. Geomicrobiol. J..

[10] Haveman S.A., Pedersen K. (2002). Microbially mediated redox processes in natural analogues for radioactive waste. J. Contam. Hydrol..

[11] Haveman S.A., Pedersen K., Ruotsalainen P. (1999). Distribution and metabolic diversity of microorganisms in deep igneous rock aquifers of Finland. Geomicrobiol. J..

[12] Dutta A., Dutta Gupta S., Gupta A., Sarkar J., Roy S., Mukherjee A., Sar P. (2018). Exploration of deep terrestrial subsurface microbiome in Late Cretaceous Deccan traps and underlying Archean basement, India. Sci. Rep..

[13] Holland G., Lollar B.S., Li L., Lacrampe-Couloume G., Slater G.F., Ballentine C.J. (2013). Deep fracture fluids isolated in the crust since the Precambrian era. Nature.

[14] Chivian D., Brodie E.L., Alm E.J., Culley D.E., Dehal P.S., DeSantis T.Z., Gihring T.M., Lapidus A., Lin L.-H.H., Lowry S.R. (2008). Environmental genomics reveals a single-species ecosystem deep within Earth. Science.

[15] Borgonie G., García-Moyano A., Litthauer D., Bert W., Bester A., van Heerden E., Möller C., Erasmus M., Onstott T.C. (2011). Nematoda from the terrestrial deep subsurface of South Africa. Nature.

[16] Baker B.J., Saw J.H., Lind A.E., Lazar C.S., Hinrichs K.-U., Teske A.P., Ettema T.J.G. (2016). Genomic inference of the metabolism of cosmopolitan subsurface Archaea, Hadesarchaea. Nat. Microbiol..

[17] Takai K., Moser D.P., DeFlaun M., Onstott T.C., Fredrickson J.K. (2001). Archaeal diversity in waters from deep South African gold mines. Appl. Environ. Microbiol..

[18] Gihring T.M., Moser D.P., Lin L.H., Davidson M., Onstott T.C., Morgan L., Milleson M., Kieft T.L., Trimarco E., Balkwill D.L. (2006). The distribution of microbial taxa in the subsurface water of the Kalahari Shield, South Africa. Geomicrobiol. J..

[19] Shimizu S., Akiyama M., Ishijima Y., Hama K., Kunimaru T., Naganuma T. (2006). Molecular characterization of microbial communities in fault-bordered aquifers in the Miocene formation of northernmost Japan. Geobiology.

[20] Fukuda A., Hagiwara H., Ishimura T., Kouduka M., Ioka S., Amano Y., Tsunogai U., Suzuki Y., Mizuno T. (2010). Geomicrobiological properties of ultra-deep granitic groundwater from the Mizunami Underground Research Laboratory (MIU), central Japan. Microb. Ecol..

[21] Purkamo L., Bomberg M., Kietäväinen R., Salavirta H., Nyyssönen M., Nuppunen-Puputti M., Ahonen L., Kukkonen I., Itävaara M. (2016). Microbial co-occurrence patterns in deep Precambrian bedrock fracture fluids. Biogeosciences.

[22] Magnabosco C., Ryan K., Lau M.C.Y., Kuloyo O., Sherwood Lollar B., Kieft T.L., van Heerden E., Onstott T.C. (2016). A metagenomic window into carbon metabolism at 3 km depth in Precambrian continental crust. ISME J..

[23] Simkus D.N., Slater G.F., Lollar B.S., Wilkie K., Kieft T.L., Magnabosco C., Lau M.C.Y.Y., Pullin M.J., Hendrickson S.B., Wommack K.E. (2016). Variations in microbial carbon sources and cycling in the deep continental subsurface. Geochim. Cosmochim. Acta.

[24] Wu X., Holmfeldt K., Hubalek V., Lundin D., Åström M., Bertilsson S., Dopson M. (2016). Microbial metagenomes from three aquifers in the Fennoscandian shield terrestrial deep biosphere reveal metabolic partitioning among populations. ISME J..

[25] Bomberg M., Nyyssönen M., Pitkänen P., Lehtinen A., Itävaara M. (2015). Active Microbial Communities Inhabit Sulphate-Methane Interphase in Deep Bedrock Fracture Fluids in Olkiluoto, Finland. Biomed Res. Int..

[26] Pedersen K. (1997). Microbial life in deep granitic rock. FEMS Microbiol. Rev..

[27] Kotelnikova S., Pedersen K. (1997). Evidence for methanogenic Archaea and homoacetogenic Bacteria in deep granitic rock aquifers. FEMS Microbiol. Rev..

[28] Hug L.A., Baker B.J., Anantharaman K., Brown C.T., Probst A.J., Castelle C.J., Butterfield C.N., Hernsdorf A.W., Amano Y., Ise K. (2016). A new view of the tree of life. Nat. Microbiol..

[29] Hug L.A., Thomas B.C., Sharon I., Brown C.T., Sharma R., Hettich R.L., Wilkins M.J., Williams K.H., Singh A., Banfield J.F. (2016). Critical biogeochemical functions in the subsurface are associated with bacteria from new phyla and little studied lineages. Environ. Microbiol..

[30] Richter A. Final Phase Launched of Drilling the World’s Deepest Geothermal Heat Wells in Otaniemi, Finland. http://www.thinkgeoenergy.com/final-phase-launched-of-drilling-the-worlds-deepest-geothermal-heat-wells-in-otaniemi-finland.

[31] Abramov O., Mojzsis S.J. (2009). Microbial habitability of the Hadean Earth during the late heavy bombardment. Nature.

[32] Ivarsson M., Lindgren P. (2010). The search for sustainable subsurface habitats on mars, and the sampling of impact ejecta. Sustainability.

[33] Rull F., Sansano A., Díaz E., Canora C.P., Moral A.G., Tato C., Colombo M., Belenguer T., Fernández M., Manfredi J.A.R. (2011). ExoMars Raman laser spectrometer for Exomars. Instruments, Methods, and Missions for Astrobiology XIV.

[34] Vago J.L., Westall F., Coates A.J., Jaumann R., Korablev O., Ciarletti V., Mitrofanov I., Josset J.L., De Sanctis M.C., Bibring J.P. (2017). Habitability on Early Mars and the Search for Biosignatures with the ExoMars Rover. Astrobiology.

[35] Osburn M.R., LaRowe D.E., Momper L.M., Amend J.P. (2014). Chemolithotrophy in the continental deep subsurface: Sanford Underground Research Facility (SURF), USA. Front. Microbiol..

[36] Colman D.R., Poudel S., Stamps B.W., Boyd E.S., Spear J.R. (2017). The deep, hot biosphere: Twenty-five years of retrospection. Proc. Natl. Acad. Sci. USA.

[37] Miettinen H., Kietäväinen R., Sohlberg E., Numminen M., Ahonen L., Itävaara M. (2015). Microbiome composition and geochemical characteristics of deep subsurface high-pressure environment, Pyhäsalmi mine Finland. Front. Microbiol..

[38] Leary P., Malin P., Saarno T., Kukkonen I. (2017). Prospects for assessing enhanced geothermal system (EGS) basement rock flow stimulation by wellbore temperature data. Energies.

[39] Pajunen M., Airo M.L., Elminen T., Niemelä R., Salmelainen J., Vaarma M., Wasenius P., Wennerström M. (2008). Construction suitability of bedrock in the Helsinki area based on the tectonic structure of the Svecofennian crust of southern Finland. Spec. Pap. Geol. Surv. Finl..

[40] Herlemann D.P., Labrenz M., Jürgens K., Bertilsson S., Waniek J.J., Andersson A.F. (2011). Transitions in bacterial communities along the 2000km salinity gradient of the Baltic Sea. ISME J..

[41] Klindworth A., Pruesse E., Schweer T., Peplies J., Quast C., Horn M., Glöckner F.O., Klindworth A., Pruesse E., Schweer T. (2013). Evaluation of general 16S ribosomal RNA gene PCR primers for classical and next-generation sequencing-based diversity studies. Nucleic Acids Res..

[42] White T.J., Bruns S., Lee S., Taylor J. (1990). Amplification and direct sequencing of fungal ribosomal RNA genes for phyologenetics.pdf. PCR Protoc. A Guid. Methods Appl..

[43] Gardes M., Bruns T.D. (1993). ITS primers with enhanced specificity for basidiomycetes-application to the identification of mycorrhizae and rusts. Mol. Ecol..

[44] Lever M.A., Torti A., Eickenbusch P., Michaud A.B., Šantl-Temkiv T., Jørgensen B.B. (2015). A modular method for the extraction of DNA and RNA, and the separation of DNA pools from diverse environmental sample types. Front. Microbiol..

[45] Ohkuma M., Kudo T. (1998). Phylogenetic analysis of the symbiotic intestinal microflora of the termite *Cryptotermes domesticus*. FEMS Microbiol. Lett..

[46] Cadillo-Quiroz H., Bräuer S., Yashiro E., Sun C., Yavitt J., Zinder S. (2006). Vertical profiles of methanogenesis and methanogens in two contrasting acidic peatlands in central New York State, USA. Environ. Microbiol..

[47] Yu Y., Lee C., Kim J., Hwang S. (2005). Group-specific primer and probe sets to detect methanogenic communities using quantitative real-time polymerase chain reaction. Biotechnol. Bioeng..

[48] Wagner M., Roger A.J., Flax J.L., Gregory A., Stahl D.A., Wagner M., Roger A.J., Flax J.L., Brusseau G.A., Stahl D.A. (1998). Phylogeny of Dissimilatory Sulfite Reductases Supports an Early Origin of Sulfate Respiration Phylogeny of Dissimilatory Sulfite Reductases Supports an Early Origin of Sulfate Respiration. J. Bacteriol..

[49] Geets J., Borremans B., Diels L., Springael D., Vangronsveld J., Van Der Lelie D., Vanbroekhoven K. (2006). DsrB gene-based DGGE for community and diversity surveys of sulfate-reducing bacteria. J. Microbiol. Methods.

[50] Steinberg L.M., Regan J.M. (2008). Phylogenetic comparison of the methanogenic communities from an acidic, oligotrophic fen and an anaerobic digester treating municipal wastewater sludge. Appl. Environ. Microbiol..

[51] López-Gutiérrez J.C., Henry S., Hallet S., Martin-Laurent F., Catroux G., Philippot L. (2004). Quantification of a novel group of nitrate-reducing bacteria in the environment by real-time PCR. J. Microbiol. Methods.

[52] Hales B.A., Edwards C., Ritchie D.A., Hall G., Pickup R.W., Saunders J.R. (1996). Isolation and identification of methanogen-specific DNA from blanket bog peat by PCR amplification and sequence analysis. Appl. Environ. Microbiol..

[53] Schloss P.D., Westcott S.L., Ryabin T., Hall J.R., Hartmann M., Hollister E.B., Lesniewski R.A., Oakley B.B., Parks D.H., Robinson C.J. (2009). Introducing mothur: Open-source, platform-independent, community-supported software for describing and comparing microbial communities. Appl. Environ. Microbiol..

[54] Purkamo L., Bomberg M., Nyyssönen M., Ahonen L., Kukkonen I., Itävaara M., Palmer K. (2017). Response of Deep Subsurface Microbial Community to Different Carbon Sources and Electron Acceptors during 2 months Incubation in Microcosms. Front. Microbiol..

[55] Nilsson R.H., Larsson K.H., Taylor A.F.S., Bengtsson-Palme J., Jeppesen T.S., Schigel D., Kennedy P., Picard K., Glöckner F.O., Tedersoo L. (2019). The UNITE database for molecular identification of fungi: Handling dark taxa and parallel taxonomic classifications. Nucleic Acids Res..

[56] Finn R.D., Cochrane G., Qureshi M., Scheremetjew M., Potter S., Mitchell A.L., Amid C., ten Hoopen P., Boland M.A., Wilkinson D.J. (2017). EBI Metagenomics in 2017: Enriching the analysis of microbial communities, from sequence reads to assemblies. Nucleic Acids Res..

[57] Bolger A.M., Lohse M., Usadel B. (2014). Trimmomatic: A flexible trimmer for Illumina sequence data. Bioinformatics.

[58] Afgan E., Baker D., van den Beek M., Blankenberg D., Bouvier D., Čech M., Chilton J., Clements D., Coraor N., Eberhard C. (2016). The Galaxy platform for accessible, reproducible and collaborative biomedical analyses: 2016 update. Nucleic Acids Res..

[59] Wood D.E., Salzberg S.L. (2014). Kraken: Ultrafast metagenomic sequence classification using exact alignments. Genome Biol..

[60] Moriya Y., Itoh M., Okuda S., Yoshizawa A.C., Kanehisa M. (2007). KAAS: An automatic genome annotation and pathway reconstruction server. Nucleic Acids Res..

[61] Kanehisa M., Sato Y., Morishima K. (2016). BlastKOALA and GhostKOALA: KEGG Tools for Functional Characterization of Genome and Metagenome Sequences. J. Mol. Biol..

[62] Li D., Liu C.M., Luo R., Sadakane K., Lam T.W. (2015). MEGAHIT: An ultra-fast single-node solution for large and complex metagenomics assembly via succinct de Bruijn graph. Bioinformatics.

[63] Louca S., Parfrey L.W., Doebeli M. (2016). Decoupling function and taxonomy in the global ocean microbiome. Science.

[64] Comeau A.M., Douglas G.M., Langille M.G.I. (2017). Microbiome Helper: A Custom and Streamlined Workflow for Microbiome Research. MSystems.

[65] Kassambara A. ggpubr: “ggplot2” Based Publication Ready Plots. R package Version 0.2. https://CRAN.R-project.org/package=ggpubr.

[66] McMurdie P.J., Holmes S. (2013). Phyloseq: An R Package for Reproducible Interactive Analysis and Graphics of Microbiome Census Data. PLoS ONE.

[67] Glud R.N., Wenzhöfer F., Middelboe M., Oguri K., Turnewitsch R., Canfield D.E., Kitazato H. (2013). High rates of microbial carbon turnover in sediments in the deepest oceanic trench on Earth. Nat. Geosci..

[68] Hernsdorf A.W., Amano Y., Miyakawa K., Ise K., Suzuki Y., Anantharaman K., Probst A., Burstein D., Thomas B.C., Banfield J.F. (2017). Potential for microbial H_2_ and metal transformations associated with novel bacteria and archaea in deep terrestrial subsurface sediments. ISME J..

[69] Breuker A., Köweker G., Blazejak A., Schippers A., Koweker G., Blazejak A., Schippers A. (2011). The deep biosphere in terrestrial sediments in the Chesapeake Bay area, Virginia, USA. Front. Microbiol..

[70] Mcmahon S., Parnell J. (2014). Weighing the deep continental biosphere. FEMS Microbiol. Ecol..

[71] Magnabosco C., Lin L.H., Dong H., Bomberg M., Ghiorse W., Stan-Lotter H., Pedersen K., Kieft T.L., van Heerden E., Onstott T.C. (2018). The biomass and biodiversity of the continental subsurface. Nat. Geosci..

[72] Jørgensen B.B. (2012). Shrinking majority of the deep biosphere. Proc. Natl. Acad. Sci. USA.

[73] Kallmeyer J., Pockalny R., Adhikari R.R., Smith D.C., D’Hondt S. (2012). From the Cover: Global distribution of microbial abundance and biomass in subseafloor sediment. Proc. Natl. Acad. Sci. USA.

[74] Bar-On Y.M., Phillips R., Milo R. (2018). The biomass distribution on Earth. Proc. Natl. Acad. Sci. USA.

[75] Fairén A.G., Davila A.F., Lim D., Bramall N., Bonaccorsi R., Zavaleta J., Uceda E.R., Stoker C., Wierzchos J., Dohm J.M. (2010). Astrobiology through the ages of Mars: The study of terrestrial analogues to understand the habitability of Mars. Astrobiology.

[76] Neveu M., Hays L.E., Voytek M.A., New M.H., Schulte M.D. (2018). The ladder of life detection. Astrobiology.

[77] Hunter M.E., Dorazio R.M., Butterfield J.S.S., Meigs-Friend G., Nico L.G., Ferrante J.A. (2017). Detection limits of quantitative and digital PCR assays and their influence in presence–absence surveys of environmental DNA. Mol. Ecol. Resour..

[78] Bogan B.W., Sullivan W.R., Kayser K.J., Derr K.D., Aldrich H.C., Paterek J.R. (2003). *Alkanindiges illinoisensis* gen. nov., sp. nov., an obligately hydrocarbonoclastic, aerobic squalane-degrading bacterium isolated from oilfield soils. Int. J. Syst. Evol. Microbiol..

[79] Pedersen K., Nilsson E., Arlinger J., Hallbeck L., O’Neill A. (2004). Distribution, diversity and activity of microorganisms in the hyper-alkaline spring waters of Maqarin in Jordan. Extremophiles.

[80] Purkamo L., Bomberg M., Kietäväinen R., Salavirta H., Nyyssönen M., Nuppunen-Puputti M., Ahonen L., Kukkonen I., Itävaara M. (2015). The keystone species of Precambrian deep bedrock biosphere belong to Burkholderiales and Clostridiales. Biogeosciences Discuss..

[81] Soares A., Edwards A., An D., Bagnoud A., Bomberg M., Budwill K., Caffrey S.M., Fields M., Gralnick J., Kadnikov V. (2019). A global perspective on microbial diversity in the terrestrial deep subsurface. bioRxiv.

[82] Hubalek V., Wu X., Eiler A., Buck M., Heim C., Dopson M., Bertilsson S., Ionescu D. (2016). Connectivity to the surface determines diversity patterns in subsurface aquifers of the Fennoscandian shield. ISME J..

[83] Sheik C.S., Reese B.K., Twing K.I., Sylvan J.B., Grim S.L., Schrenk M.O., Sogin M.L., Colwell F.S. (2018). Identification and removal of contaminant sequences from ribosomal gene databases: Lessons from the Census of Deep Life. Front. Microbiol..

[84] Bomberg M., Raulio M., Jylhä S., Mueller C.W., Höschen C., Rajala P., Purkamo L., Kietäväinen R., Ahonen L., Itävaara M. (2017). CO_2_ and carbonate as substrate for the activation of the microbial community in 180 m deep bedrock fracture fluid of Outokumpu Deep Drill Hole, Finland. AIMS Microbiol..

[85] Moore E.R.B., Tindall B.J., Martins Dos Santos V.A.P., Pieper D.H., Ramos J., Palleroni N.J., Dworkin M., Falkow S., Rosenberg E., Schleifer K.S., Stackebrandt E. (2006). Nonmedical: Pseudomonas. The Prokaryotes.

[86] Teixeira L.M., Merquior V.L.C., Rosenberg E., DeLong E.F., Lory S., Stackebrandt E., Thompson F. (2014). The Family Moraxellaceae. The Prokaryotes.

[87] Palleroni N.J., Starr M.P., Stolp H., Trüper H.G., Balows A., Schlegel H.G. (1981). Introduction to the Family Pseudomonadaceae. The Prokaryotes.

[88] Moser D.P., Gihring T.M., Brockman F.J., Fredrickson J.K., Balkwill D.L., Dollhopf M.E., Lollar B.S., Pratt L.M., Boice E., Southam G. (2005). *Desulfotomaculum* and *Methanobacterium* spp. dominate a 4- to 5-kilometer-deep fault. Appl. Environ. Microbiol..

[89] Purkamo L., Bomberg M., Nyyssönen M., Kukkonen I., Ahonen L., Itävaara M. (2015). Heterotrophic Communities Supplied by Ancient Organic Carbon Predominate in Deep Fennoscandian Bedrock Fluids. Microb. Ecol..

[90] Comolli L.R., Baker B.J., Downing K.H., Siegerist C.E., Banfield J.F. (2009). Three-dimensional analysis of the structure and ecology of a novel, ultra-small archaeon. ISME J..

[91] Herrmann M., Wegner C.E., Taubert M., Geesink P., Lehmann K., Yan L., Lehmann R., Totsche K.U., Küsel K. (2019). Predominance of Cand. Patescibacteria in groundwater is caused by their preferential mobilization from soils and flourishing under oligotrophic conditions. Front. Microbiol..

[92] Jousset A., Bienhold C., Chatzinotas A., Gallien L., Gobet A., Kurm V., Küsel K., Rillig M.C., Rivett D.W., Salles J.F. (2017). Where less may be more: How the rare biosphere pulls ecosystems strings. ISME J..

[93] Nuppunen-Puputti M., Purkamo L., Kietäväinen R., Nyyssönen M., Itävaara M., Ahonen L., Kukkonen I., Bomberg M. (2018). Rare biosphere archaea assimilate acetate in Precambrian terrestrial subsurface at 2.2 km depth. Geosciences.

[94] Sohlberg E., Bomberg M., Miettinen H., Nyyssönen M., Salavirta H., Vikman M., Itävaara M. (2015). Revealing the unexplored fungal communities in deep groundwater of crystalline bedrock fracture zones in Olkiluoto, Finland. Front. Microbiol..

[95] Ivarsson M., Bengtson S., Drake H., Francis W. (2017). Fungi in Deep Subsurface Environments.

[96] Rummel J.D., Beaty D.W., Jones M.A., Bakermans C., Barlow N.G., Boston P.J., Chevrier V.F., Clark B.C., De Vera J.P.P., Gough R.V. (2014). A new analysis of mars “Special Regions”: Findings of the second MEPAG special regions science analysis group (SR-SAG2). Astrobiology.

[97] Seto M., Noguchi K., Van Cappellen P. (2019). Potential for Aerobic Methanotrophic Metabolism on Mars. Astrobiology.

[98] Onstott T.C., Ehlmann B.L., Sapers H., Coleman M., Ivarsson M., Marlow J.J., Neubeck A., Niles P. (2019). Paleo-Rock-Hosted Life on Earth and the Search on Mars: A Review and Strategy for Exploration. Astrobiology.

[99] Eigenbrode J.L., Summons R.E., Steele A., Freissinet C., Millan M., Navarro-González R., Sutter B., McAdam A.C., Franz H.B., Glavin D.P. (2018). Organic matter preserved in 3-billion-year-old mudstones at Gale crater, Mars. Science.

[100] Waite J.H., Combi M.R., Ip W.H., Cravens T.E., McNutt R.L., Kasprzak W., Yelle R., Luhmann J., Niemann H., Gell D. (2006). Cassini ion and neutral mass spectrometer: Enceladus plume composition and structure. Science.

[101] Postberg F., Khawaja N., Abel B., Choblet G., Glein C.R., Gudipati M.S., Henderson B.L., Hsu H.W., Kempf S., Klenner F. (2018). Macromolecular organic compounds from the depths of Enceladus. Nature.

[102] Berg I.A. (2011). Ecological aspects of the distribution of different autotrophic CO_2_ fixation pathways. Appl. Environ. Microbiol..

[103] Stern J.C., Sutter B., Freissinet C., Navarro-González R., McKay C.P., Archer P.D., Buch A., Brunner A.E., Coll P., Eigenbrode J.L. (2015). Evidence for indigenous nitrogen in sedimentary and aeolian deposits from the Curiosity rover investigations at Gale crater, Mars. Proc. Natl. Acad. Sci. USA.

[104] Franz H.B., Trainer M.G., Malespin C.A., Mahaffy P.R., Atreya S.K., Becker R.H., Benna M., Conrad P.G., Eigenbrode J.L., Freissinet C. (2017). Initial SAM calibration gas experiments on Mars: Quadrupole mass spectrometer results and implications. Planet. Space Sci..

[105] Könneke M., Bernhard A.E., de la Torre J.R., Walker C.B., Waterbury J.B., Stahl D. (2005). A Isolation of an autotrophic ammonia-oxidizing marine archaeon. Nature.

[106] Qin W., Heal K.R., Ramdasi R., Kobelt J.N., Martens-Habbena W., Bertagnolli A.D., Amin S.A., Walker C.B., Urakawa H., Könneke M. (2017). *Nitrosopumilus maritimus* gen. nov., sp. nov., *Nitrosopumilus cobalaminigenes* sp. nov., *Nitrosopumilus oxyclinae* sp. nov., and *Nitrosopumilus ureiphilus* sp. nov., four marine ammoniaoxidizing archaea of the phylum thaumarchaeo. Int. J. Syst. Evol. Microbiol..

[107] Shen J., Zerkle A., Claire M., Stueeken E. (2019). Atmospheric Nitrate as a Potential Nutrient for Life on Mars. Extremophiles.

[108] Cockell C.S. (2014). Trajectories of martian habitability. Astrobiology.

[109] Gudelj I., Beardmore R.E., Arkin S.S., Maclean R.C. (2007). Constraints on microbial metabolism drive evolutionary diversification in homogeneous environments. J. Evol. Biol..

[110] Fernandez-Gonzalez N., Huber J.A., Vallino J.J. (2016). Microbial Communities are Well Adapted to Disturbances in Energy Input Nuria Fernandez-Gonzalez. MSystems.

[111] Salter S.J., Cox M.J., Turek E.M., Calus S.T., Cookson W.O., Moffatt M.F., Turner P., Parkhill J., Loman N.J., Walker A.W. (2014). Reagent and laboratory contamination can critically impact sequence-based microbiome analyses. BMC Biol..

[112] Carr C.E., Mojarro A., Tani J., Bhattaru S.A., Zuber M.T., Doebler R., Brown M., Herrington K., Talbot R., Fuller C.W. Advancing the search for extra-terrestrial genomes. Proceedings of the 2016 IEEE Aerospace Conference.

[113] Carr C.E., Mojarro A., Hachey J., Saboda K., Tani J., Bhattaru S.A., Smith A., Pontefract A., Zuber M.T., Doebler R. Towards in situ sequencing for life detection. Proceedings of the 2017 IEEE Aerospace Conference.

