# Supplementary material for: Ultradeep Microbial Communities at 4.4 km within Crystalline Bedrock: Implications for Habitability in a Planetary Context
Source: Life (Basel). 2020 Jan 4;10(1):2. doi: 10.3390/life10010002 (PMC7175195; doi:10.3390/life10010002)
Supplement: Supplementary file 1 [file life-10-00002-s001.zip › SupplementaryFigure1_revised.html]

Javascript must be enabled to view this page.

members
count
unassigned
rank

0

node0.members.0.js
65288
435

node1.members.0.js
63255
1560
superkingdom

10
phylum

10
class

10
order

10
family

10
genus

node7.members.0.js
10
species

node8.members.0.js
687
120
phylum

10
class

10
order

10
genus

node12.members.0.js
7
species

node13.members.0.js
3
species

69
order

69
family

69
genus

node17.members.0.js
69
58
species

node18.members.0.js
10
subspecies

node19.members.0.js
1
subspecies

116
order

node21.members.0.js
104
24
family

node22.members.0.js
47
16
genus

node23.members.0.js
30
species

node24.members.0.js
1
species

19
genus

node26.members.0.js
19
species

14
genus

node28.members.0.js
4
species

node29.members.0.js
10
species

12
family

2
genus

node32.members.0.js
2
species

10
genus

node34.members.0.js
10
species

372
subclass

160
order

17
genus

node38.members.0.js
17
species

22
genus

node40.members.0.js
22
species

37
genus

node42.members.0.js
37
species

14
genus

node44.members.0.js
14
species

28
genus

node46.members.0.js
22
species

node47.members.0.js
6
species

14
genus

node49.members.0.js
14
species

28
genus

node51.members.0.js
28
species

node52.members.0.js
212
17
order

56
genus

node54.members.0.js
56
species

node55.members.0.js
27
20
genus

node56.members.0.js
7
species

node57.members.0.js
6
2
genus

node58.members.0.js
4
species

8
genus

node60.members.0.js
8
species

20
genus

node62.members.0.js
18
species

node63.members.0.js
2
species

12
genus

node65.members.0.js
12
species

node66.members.0.js
34
13
genus

node67.members.0.js
11
species

node68.members.0.js
2
species

node69.members.0.js
2
species

node70.members.0.js
4
species

node71.members.0.js
2
species

30
genus

node73.members.0.js
30
species

2
genus

node75.members.0.js
2
species

6
phylum

node77.members.0.js
6
species

554
phylum

node79.members.0.js
554
12
class

15
order

15
family

node82.members.0.js
15
4
genus

node83.members.0.js
2
species

node84.members.0.js
6
species

node85.members.0.js
3
species

473
order

node87.members.0.js
473
6
family

2
genus

node89.members.0.js
2
species

node90.members.0.js
465
17
genus

node91.members.0.js
2
species

node92.members.0.js
9
species

node93.members.0.js
39
species

node94.members.0.js
16
species

node95.members.0.js
57
18
species

node96.members.0.js
39
subspecies

node97.members.0.js
170
species

node98.members.0.js
1
species

2
species

node100.members.0.js
2
subspecies

node101.members.0.js
23
species

node102.members.0.js
4
species

node103.members.0.js
13
species

node104.members.0.js
102
species

node105.members.0.js
10
species

54
order

54
family

node108.members.0.js
42
2
genus

node109.members.0.js
40
species

12
genus

node111.members.0.js
2
species

node112.members.0.js
10
species

7
phylum

7
class

7
order

3
family

node117.members.0.js
3
genus

4
family

4
genus

node120.members.0.js
4
species

141
phylum

141
class

141
order

89
family

4
genus

node126.members.0.js
4
species

85
genus

node128.members.0.js
85
40
species

node129.members.0.js
1
subspecies

node130.members.0.js
12
subspecies

node131.members.0.js
32
subspecies

52
family

52
genus

node134.members.0.js
52
species

5
phylum

node136.members.0.js
5
species

9
phylum

9
class

9
order

9
family

9
genus

node142.members.0.js
9
species

6
phylum

6
class

6
order

6
family

1
genus

node148.members.0.js
1
species

3
genus

node150.members.0.js
3
species

2
genus

node152.members.0.js
2
species

19
phylum

19
class

3
order

node156.members.0.js
3
family

16
order

16
family

16
genus

node160.members.0.js
16
species

node161.members.0.js
23825
42
phylum

406
class

32
order

node164.members.0.js
32
2
family

1
genus

node166.members.0.js
1
species

12
genus

node168.members.0.js
12
species

11
genus

node170.members.0.js
11
species

6
genus

node172.members.0.js
6
species

374
order

4
family

4
genus

node176.members.0.js
4
species

370
family

364
genus

node179.members.0.js
364
species

6
genus

node181.members.0.js
6
species

397
class

397
order

397
family

397
genus

node186.members.0.js
397
species

53
class

53
order

53
family

10
genus

node191.members.0.js
10
species

43
genus

node193.members.0.js
43
species

node194.members.0.js
22920
489
class

76
order

76
family

76
genus

node198.members.0.js
76
species

29
order

29
family

29
genus

node202.members.0.js
29
species

441
order

441
family

441
genus

node206.members.0.js
441
species

4
order

4
family

4
genus

node210.members.0.js
4
species

487
order

node212.members.0.js
487
19
family

node213.members.0.js
339
24
genus

node214.members.0.js
60
species

node215.members.0.js
63
species

node216.members.0.js
77
species

node217.members.0.js
115
species

38
genus

node219.members.0.js
38
species

node220.members.0.js
51
45
genus

node221.members.0.js
1
species

node222.members.0.js
5
species

node223.members.0.js
40
7
genus

node224.members.0.js
20
species

node225.members.0.js
13
species

16
genus

node227.members.0.js
16
species

59
order

59
family

59
genus

node231.members.0.js
59
species

149
order

149
family

node234.members.0.js
149
21
genus

node235.members.0.js
10
species

node236.members.0.js
43
species

node237.members.0.js
13
species

node238.members.0.js
43
species

node239.members.0.js
19
species

222
order

222
family

node242.members.0.js
92
7
genus

node243.members.0.js
50
species

node244.members.0.js
9
species

node245.members.0.js
7
4
species

node246.members.0.js
1
subspecies

node247.members.0.js
2
subspecies

node248.members.0.js
6
species

2
species

node250.members.0.js
2
subspecies

node251.members.0.js
9
species

node252.members.0.js
2
species

130
genus

node254.members.0.js
130
species

node255.members.0.js
3360
81
order

683
family

node257.members.0.js
683
51
genus

node258.members.0.js
150
species

node259.members.0.js
1
species

node260.members.0.js
29
species

node261.members.0.js
7
species

node262.members.0.js
23
species

node263.members.0.js
252
species

node264.members.0.js
42
species

node265.members.0.js
13
species

node266.members.0.js
22
species

node267.members.0.js
29
species

node268.members.0.js
2
species

node269.members.0.js
15
species

node270.members.0.js
21
species

node271.members.0.js
26
species

node272.members.0.js
427
5
family

node273.members.0.js
92
1
genus

node274.members.0.js
47
species

node275.members.0.js
44
species

node276.members.0.js
330
57
genus

node277.members.0.js
93
species

node278.members.0.js
84
species

node279.members.0.js
42
species

node280.members.0.js
16
species

node281.members.0.js
38
species

1945
family

node283.members.0.js
1937
489
genus

node284.members.0.js
6
species

node285.members.0.js
2
species

node286.members.0.js
17
species

node287.members.0.js
61
56
species

node288.members.0.js
5
subspecies

node289.members.0.js
1
species

node290.members.0.js
175
species

node291.members.0.js
5
species

node292.members.0.js
91
species

node293.members.0.js
145
species

node294.members.0.js
370
species

node295.members.0.js
12
species

node296.members.0.js
82
72
species

node297.members.0.js
10
subspecies

node298.members.0.js
188
species

node299.members.0.js
151
species

node300.members.0.js
26
species

node301.members.0.js
53
species

node302.members.0.js
1
species

node303.members.0.js
53
species

node304.members.0.js
7
species

node305.members.0.js
2
species

8
genus

node307.members.0.js
8
species

35
family

35
genus

node310.members.0.js
35
species

16
family

16
genus

node313.members.0.js
16
species

173
family

node315.members.0.js
173
7
genus

node316.members.0.js
38
species

node317.members.0.js
82
species

node318.members.0.js
46
species

170
order

19
family

19
genus

node322.members.0.js
19
species

71
family

13
genus

node325.members.0.js
13
species

node326.members.0.js
58
1
genus

42
species

node328.members.0.js
42
subspecies

node329.members.0.js
15
species

80
family

80
genus

node332.members.0.js
80
species

495
order

node334.members.0.js
495
12
family

47
genus

node336.members.0.js
47
species

node337.members.0.js
436
80
genus

33
species

node339.members.0.js
33
subspecies

node340.members.0.js
32
species

node341.members.0.js
2
species

node342.members.0.js
31
species

node343.members.0.js
9
species

node344.members.0.js
20
species

node345.members.0.js
30
species

node346.members.0.js
15
species

node347.members.0.js
26
species

node348.members.0.js
55
species

node349.members.0.js
1
species

node350.members.0.js
37
species

node351.members.0.js
6
species

24
species

node353.members.0.js
24
subspecies

node354.members.0.js
18
species

node355.members.0.js
17
species

node356.members.0.js
14005
3
order

node357.members.0.js
252
2
family

184
genus

node359.members.0.js
184
species

66
genus

node361.members.0.js
66
species

13750
family

33
genus

node364.members.0.js
33
species

node365.members.0.js
13717
63
genus

node366.members.0.js
69
species

node367.members.0.js
21
species

node368.members.0.js
14
species

14
species

node370.members.0.js
14
subspecies

node371.members.0.js
13536
13508
species

node372.members.0.js
28
subspecies

269
order

node374.members.0.js
269
9
family

85
genus

node376.members.0.js
85
species

120
genus

node378.members.0.js
120
species

55
genus

node380.members.0.js
55
species

472
order

node382.members.0.js
472
14
family

139
genus

node384.members.0.js
139
species

node385.members.0.js
137
9
genus

node386.members.0.js
50
species

node387.members.0.js
78
species

46
genus

node389.members.0.js
46
species

69
genus

node391.members.0.js
69
species

22
genus

node393.members.0.js
22
species

45
genus

node395.members.0.js
45
species

node396.members.0.js
2177
49
order

24
family

24
genus

node399.members.0.js
24
species

node400.members.0.js
1269
6
family

8
genus

node402.members.0.js
8
species

344
genus

node404.members.0.js
96
species

node405.members.0.js
248
species

75
genus

node407.members.0.js
75
species

763
genus

node409.members.0.js
763
species

node410.members.0.js
73
6
genus

node411.members.0.js
11
species

node412.members.0.js
13
species

node413.members.0.js
23
species

node414.members.0.js
14
species

node415.members.0.js
6
species

node416.members.0.js
418
14
family

36
genus

node418.members.0.js
36
8
species

node419.members.0.js
8
subspecies

node420.members.0.js
20
subspecies

278
genus

node422.members.0.js
278
99
species

node423.members.0.js
54
subspecies

node424.members.0.js
36
subspecies

node425.members.0.js
89
subspecies

90
genus

node427.members.0.js
90
species

28
family

28
genus

node430.members.0.js
28
species

41
family

41
genus

node433.members.0.js
41
species

74
family

29
genus

node436.members.0.js
29
species

45
genus

node438.members.0.js
45
species

88
family

88
genus

node441.members.0.js
88
species

1
genus

node443.members.0.js
1
species

104
family

node445.members.0.js
104
2
genus

node446.members.0.js
34
species

node447.members.0.js
44
species

node448.members.0.js
24
species

81
family

81
genus

node451.members.0.js
81
species

7
class

7
order

7
suborder

7
family

7
genus

node457.members.0.js
7
species

63
superphylum

51
phylum

51
class

51
order

4
family

1
genus

node464.members.0.js
1
species

3
genus

node466.members.0.js
3
species

node467.members.0.js
34
1
family

33
genus

node469.members.0.js
33
species

13
family

13
genus

node472.members.0.js
13
species

12
phylum

9
class

3
order

3
family

3
genus

node478.members.0.js
3
species

6
order

6
family

6
genus

node482.members.0.js
6
species

3
class

3
order

3
family

3
genus

node487.members.0.js
3
species

node488.members.0.js
24455
615
phylum

node489.members.0.js
732
9
subphylum

node490.members.0.js
81
1
class

2
order

2
family

2
genus

node494.members.0.js
2
species

73
order

62
family

node497.members.0.js
2
genus

33
genus

node499.members.0.js
1
species

node500.members.0.js
7
species

node501.members.0.js
25
species

3
genus

node503.members.0.js
3
species

24
genus

node505.members.0.js
23
species

node506.members.0.js
1
species

11
family

7
genus

node509.members.0.js
7
species

node510.members.0.js
4
1
genus

node511.members.0.js
2
species

node512.members.0.js
1
species

3
genus

node514.members.0.js
3
species

2
genus

node516.members.0.js
2
species

node517.members.0.js
642
43
class

42
order

7
family

7
genus

node521.members.0.js
2
species

node522.members.0.js
5
species

35
family

node524.members.0.js
35
7
genus

node525.members.0.js
3
species

node526.members.0.js
2
species

node527.members.0.js
11
species

node528.members.0.js
2
species

node529.members.0.js
5
species

node530.members.0.js
2
species

node531.members.0.js
3
species

34
order

4
family

4
genus

node535.members.0.js
4
species

30
family

1
genus

node538.members.0.js
1
species

29
genus

node540.members.0.js
29
species

node541.members.0.js
287
10
order

63
suborder

63
family

node544.members.0.js
63
genus

74
suborder

74
family

node547.members.0.js
74
genus

node548.members.0.js
140
2
suborder

62
family

node550.members.0.js
62
genus

18
family

node552.members.0.js
18
genus

node553.members.0.js
58
2
family

node554.members.0.js
15
genus

node555.members.0.js
41
genus

117
order

1
family

1
genus

node559.members.0.js
1
species

9
family

9
genus

node562.members.0.js
9
species

107
family

35
genus

node565.members.0.js
35
species

node566.members.0.js
72
4
genus

node567.members.0.js
8
species

node568.members.0.js
7
species

node569.members.0.js
2
species

node570.members.0.js
3
species

node571.members.0.js
18
species

node572.members.0.js
12
species

node573.members.0.js
1
species

node574.members.0.js
17
species

5
order

5
family

5
genus

node578.members.0.js
5
species

100
order

node580.members.0.js
22
2
family

14
genus

node582.members.0.js
14
species

2
genus

node584.members.0.js
2
species

4
genus

node586.members.0.js
4
species

78
family

58
genus

node589.members.0.js
58
species

6
genus

node591.members.0.js
6
species

14
genus

node593.members.0.js
14
species

9
order

1
family

1
genus

node597.members.0.js
1
species

8
family

8
genus

node600.members.0.js
8
species

5
order

5
family

5
genus

node604.members.0.js
5
species

node605.members.0.js
10661
182
class

node606.members.0.js
464
4
order

node607.members.0.js
118
2
family

19
genus

node609.members.0.js
19
species

4
genus

node611.members.0.js
4
species

3
genus

node613.members.0.js
3
species

node614.members.0.js
75
71
genus

node615.members.0.js
1
species

node616.members.0.js
3
species

15
genus

node618.members.0.js
15
species

node619.members.0.js
342
4
family

node620.members.0.js
138
7
genus

node621.members.0.js
60
species

node622.members.0.js
71
species

42
genus

node624.members.0.js
42
species

node625.members.0.js
50
2
genus

node626.members.0.js
23
species

node627.members.0.js
25
species

10
genus

node629.members.0.js
10
species

98
genus

node631.members.0.js
70
species

node632.members.0.js
28
species

node633.members.0.js
18
2
order

16
family

node635.members.0.js
8
species

8
genus

node637.members.0.js
8
species

78
genus

node639.members.0.js
78
species

4
order

4
family

4
genus

node643.members.0.js
4
species

node644.members.0.js
686
7
order

23
family

23
genus

node647.members.0.js
23
species

node648.members.0.js
656
31
family

63
genus

node650.members.0.js
63
species

node651.members.0.js
127
1
genus

node652.members.0.js
73
species

node653.members.0.js
53
species

node654.members.0.js
179
26
genus

node655.members.0.js
65
species

node656.members.0.js
39
species

node657.members.0.js
49
species

node658.members.0.js
256
6
genus

node659.members.0.js
138
species

node660.members.0.js
112
species

node661.members.0.js
8360
201
order

19
family

node663.members.0.js
19
8
genus

node664.members.0.js
1
species

node665.members.0.js
10
species

node666.members.0.js
59
4
family

41
genus

node668.members.0.js
41
species

node669.members.0.js
14
4
genus

node670.members.0.js
10
species

44
family

44
genus

node673.members.0.js
44
species

288
family

node675.members.0.js
262
57
genus

node676.members.0.js
34
species

node677.members.0.js
76
species

node678.members.0.js
46
species

node679.members.0.js
49
species

26
genus

node681.members.0.js
26
species

277
family

4
genus

node684.members.0.js
4
species

node685.members.0.js
265
2
genus

node686.members.0.js
96
species

node687.members.0.js
49
species

node688.members.0.js
118
species

8
genus

node690.members.0.js
8
species

18
family

18
genus

node693.members.0.js
18
species

node694.members.0.js
937
35
family

node695.members.0.js
394
34
genus

node696.members.0.js
36
species

node697.members.0.js
143
species

node698.members.0.js
181
species

node699.members.0.js
7
1
genus

node700.members.0.js
2
species

node701.members.0.js
4
species

node702.members.0.js
215
2
genus

node703.members.0.js
149
species

node704.members.0.js
43
species

node705.members.0.js
21
species

node706.members.0.js
286
17
genus

node707.members.0.js
80
species

node708.members.0.js
114
species

node709.members.0.js
66
species

node710.members.0.js
9
species

node711.members.0.js
296
3
family

126
genus

node713.members.0.js
126
species

113
genus

node715.members.0.js
113
species

54
genus

node717.members.0.js
54
species

939
family

node719.members.0.js
939
62
genus

node720.members.0.js
63
species

node721.members.0.js
146
species

node722.members.0.js
112
species

node723.members.0.js
150
species

node724.members.0.js
406
species

node725.members.0.js
5227
76
family

72
genus

node727.members.0.js
32
species

node728.members.0.js
40
species

832
genus

node730.members.0.js
832
species

70
genus

node732.members.0.js
70
species

node733.members.0.js
4177
547
genus

node734.members.0.js
256
species

node735.members.0.js
178
species

node736.members.0.js
842
species

node737.members.0.js
1260
species

node738.members.0.js
885
species

node739.members.0.js
209
species

55
family

28
genus

28
species

node743.members.0.js
28
subspecies

27
genus

node745.members.0.js
27
species

12
order

12
family

12
genus

node749.members.0.js
10
species

node750.members.0.js
2
species

501
order

node752.members.0.js
501
7
family

50
genus

node754.members.0.js
50
species

node755.members.0.js
319
35
genus

node756.members.0.js
69
species

node757.members.0.js
108
species

node758.members.0.js
107
species

35
genus

node760.members.0.js
35
species

90
genus

node762.members.0.js
90
species

1
genus

node764.members.0.js
1
species

node765.members.0.js
355
2
order

15
family

1
genus

node768.members.0.js
1
species

14
genus

node770.members.0.js
14
species

node771.members.0.js
338
12
family

20
genus

node773.members.0.js
20
species

42
genus

node775.members.0.js
14
species

node776.members.0.js
28
species

77
genus

node778.members.0.js
50
species

node779.members.0.js
27
species

3
genus

node781.members.0.js
3
species

121
genus

node783.members.0.js
26
species

node784.members.0.js
95
species

31
genus

node786.members.0.js
31
species

3
genus

node788.members.0.js
3
species

10
genus

node790.members.0.js
6
species

node791.members.0.js
4
species

12
genus

node793.members.0.js
6
species

node794.members.0.js
6
species

2
genus

node796.members.0.js
2
species

5
genus

node798.members.0.js
5
species

node799.members.0.js
6096
156
class

323
order

node801.members.0.js
323
2
family

9
genus

node803.members.0.js
9
species

4
genus

node805.members.0.js
4
species

node806.members.0.js
2
1
genus

node807.members.0.js
1
species

1
genus

node809.members.0.js
1
species

node810.members.0.js
50
1
genus

node811.members.0.js
46
species

node812.members.0.js
3
species

29
genus

node814.members.0.js
29
species

226
genus

node816.members.0.js
193
species

node817.members.0.js
2
species

node818.members.0.js
31
species

24
order

node820.members.0.js
24
1
family

1
genus

node822.members.0.js
1
species

15
genus

node824.members.0.js
3
species

node825.members.0.js
12
10
species

node826.members.0.js
2
subspecies

7
genus

node828.members.0.js
7
species

6
genus

node830.members.0.js
6
species

72
order

node832.members.0.js
72
1
family

9
genus

node834.members.0.js
9
species

56
genus

node836.members.0.js
56
species

6
genus

node838.members.0.js
6
species

17
order

7
family

7
genus

node842.members.0.js
7
species

10
family

10
genus

7
species

node846.members.0.js
7
subspecies

node847.members.0.js
3
species

265
order

8
family

8
genus

node851.members.0.js
8
species

42
family

42
genus

node854.members.0.js
1
species

node855.members.0.js
41
species

37
family

node857.members.0.js
37
2
genus

node858.members.0.js
2
species

node859.members.0.js
22
species

node860.members.0.js
3
species

node861.members.0.js
2
species

node862.members.0.js
6
species

34
family

34
genus

node865.members.0.js
34
species

1
family

1
genus

node868.members.0.js
1
species

143
family

39
genus

node871.members.0.js
39
species

node872.members.0.js
100
1
genus

node873.members.0.js
65
species

node874.members.0.js
34
species

4
genus

node876.members.0.js
4
species

591
order

node878.members.0.js
591
9
family

node879.members.0.js
246
22
genus

node880.members.0.js
4
species

1
species

node882.members.0.js
1
subspecies

1
species

node884.members.0.js
1
subspecies

node885.members.0.js
1
species

node886.members.0.js
217
species

220
genus

node888.members.0.js
220
species

41
genus

node890.members.0.js
14
species

node891.members.0.js
27
species

5
genus

node893.members.0.js
5
species

52
genus

node895.members.0.js
52
species

18
genus

node897.members.0.js
18
species

1698
order

node899.members.0.js
1698
413
family

node900.members.0.js
17
15
genus

node901.members.0.js
2
species

node902.members.0.js
6
species

7
genus

node904.members.0.js
7
species

333
genus

node906.members.0.js
20
species

node907.members.0.js
31
species

node908.members.0.js
206
species

node909.members.0.js
12
species

node910.members.0.js
17
species

node911.members.0.js
47
27
species

node912.members.0.js
13
subspecies

node913.members.0.js
7
subspecies

node914.members.0.js
29
6
genus

node915.members.0.js
1
species

node916.members.0.js
17
species

node917.members.0.js
4
species

node918.members.0.js
1
species

node919.members.0.js
101
19
genus

node920.members.0.js
41
34
species

node921.members.0.js
7
subspecies

node922.members.0.js
41
species

3
genus

node924.members.0.js
3
species

2
genus

node926.members.0.js
2
species

2
genus

node928.members.0.js
2
species

64
genus

64
species

node931.members.0.js
64
subspecies

6
genus

node933.members.0.js
1
species

5
species

node935.members.0.js
5
subspecies

node936.members.0.js
51
2
genus

node937.members.0.js
1
species

node938.members.0.js
47
species

node939.members.0.js
1
species

node940.members.0.js
83
6
genus

node941.members.0.js
10
species

node942.members.0.js
62
species

node943.members.0.js
5
species

node944.members.0.js
28
species

12
genus

node946.members.0.js
12
species

370
genus

node948.members.0.js
370
species

node949.members.0.js
45
2
genus

node950.members.0.js
24
species

node951.members.0.js
4
species

node952.members.0.js
1
species

node953.members.0.js
14
species

node954.members.0.js
13
4
genus

node955.members.0.js
2
species

7
species

node957.members.0.js
7
subspecies

8
genus

node959.members.0.js
8
species

12
genus

node961.members.0.js
12
species

node962.members.0.js
32
2
genus

node963.members.0.js
2
species

node964.members.0.js
28
species

12
genus

12
species

node967.members.0.js
12
subspecies

33
genus

node969.members.0.js
33
species

16
genus

node971.members.0.js
16
species

157
order

2
family

2
genus

node975.members.0.js
2
species

129
family

25
genus

node978.members.0.js
25
species

64
genus

node980.members.0.js
64
species

26
genus

node982.members.0.js
26
species

14
genus

node984.members.0.js
14
species

7
family

7
genus

node987.members.0.js
4
species

node988.members.0.js
3
species

19
family

18
genus

node991.members.0.js
1
species

node992.members.0.js
17
species

1
genus

node994.members.0.js
1
species

2516
order

node996.members.0.js
1656
5
family

34
subfamily

34
genus

node999.members.0.js
34
species

node1000.members.0.js
1617
207
genus

node1001.members.0.js
12
species

node1002.members.0.js
116
species

node1003.members.0.js
2
species

node1004.members.0.js
14
species

node1005.members.0.js
26
species

node1006.members.0.js
30
species

node1007.members.0.js
26
species

node1008.members.0.js
17
species

8
species

node1010.members.0.js
8
subspecies

node1011.members.0.js
219
species

node1012.members.0.js
39
species

node1013.members.0.js
33
species

node1014.members.0.js
180
species

node1015.members.0.js
49
species

node1016.members.0.js
147
species

node1017.members.0.js
399
species

node1018.members.0.js
41
species

node1019.members.0.js
29
species

node1020.members.0.js
23
species

node1021.members.0.js
860
16
family

node1022.members.0.js
209
32
genus

node1023.members.0.js
50
species

node1024.members.0.js
28
species

node1025.members.0.js
11
species

node1026.members.0.js
88
species

67
genus

67
subgenus

node1029.members.0.js
67
species

node1030.members.0.js
568
71
genus

node1031.members.0.js
1
species

node1032.members.0.js
14
species

node1033.members.0.js
367
species

node1034.members.0.js
115
species

node1035.members.0.js
1
species

59
order

59
family

6
genus

node1039.members.0.js
6
species

node1040.members.0.js
43
18
genus

node1041.members.0.js
3
species

node1042.members.0.js
6
species

node1043.members.0.js
6
species

node1044.members.0.js
10
species

10
genus

node1046.members.0.js
10
species

33
order

33
family

15
genus

node1050.members.0.js
15
species

2
genus

node1052.members.0.js
2
species

16
genus

node1054.members.0.js
16
species

node1055.members.0.js
2
species

134
order

node1057.members.0.js
100
19
family

26
genus

node1059.members.0.js
26
species

7
genus

node1061.members.0.js
7
species

node1062.members.0.js
43
13
genus

node1063.members.0.js
24
species

node1064.members.0.js
3
species

node1065.members.0.js
3
species

5
genus

node1067.members.0.js
5
species

node1068.members.0.js
34
2
family

17
genus

node1070.members.0.js
17
species

5
genus

node1072.members.0.js
5
species

2
genus

node1074.members.0.js
2
species

8
genus

node1076.members.0.js
8
species

27
order

4
family

node1079.members.0.js
4
2
genus

1
species

node1081.members.0.js
1
subspecies

1
species

node1083.members.0.js
1
subspecies

23
family

12
genus

node1086.members.0.js
12
species

11
genus

node1088.members.0.js
10
species

node1089.members.0.js
1
species

15
order

15
family

15
genus

node1093.members.0.js
7
species

node1094.members.0.js
4
species

node1095.members.0.js
4
species

node1096.members.0.js
6351
94
class

node1097.members.0.js
2
genus

17
order

17
family

node1100.members.0.js
17
9
genus

node1101.members.0.js
7
species

node1102.members.0.js
1
species

node1103.members.0.js
5776
273
order

node1104.members.0.js
263
12
family

2
genus

node1106.members.0.js
2
species

node1107.members.0.js
92
23
genus

node1108.members.0.js
18
species

node1109.members.0.js
20
species

node1110.members.0.js
31
species

2
genus

node1112.members.0.js
2
species

3
genus

node1114.members.0.js
3
species

152
genus

node1116.members.0.js
152
species

226
genus

node1118.members.0.js
226
species

117
genus

node1120.members.0.js
117
species

185
family

9
genus

node1123.members.0.js
9
species

77
genus

node1125.members.0.js
77
species

74
genus

node1127.members.0.js
74
species

25
genus

node1129.members.0.js
25
species

node1130.members.0.js
65
9
genus

node1131.members.0.js
53
species

node1132.members.0.js
3
species

168
genus

node1134.members.0.js
168
species

node1135.members.0.js
2116
108
family

39
genus

node1137.members.0.js
39
species

node1138.members.0.js
695
175
genus

node1139.members.0.js
59
species

node1140.members.0.js
22
species

node1141.members.0.js
14
species

node1142.members.0.js
47
species

node1143.members.0.js
25
species

node1144.members.0.js
27
species

node1145.members.0.js
9
species

node1146.members.0.js
5
species

node1147.members.0.js
7
species

node1148.members.0.js
15
species

node1149.members.0.js
3
species

node1150.members.0.js
4
species

node1151.members.0.js
29
species

node1152.members.0.js
60
species

node1153.members.0.js
38
species

node1154.members.0.js
26
species

node1155.members.0.js
14
species

node1156.members.0.js
27
species

node1157.members.0.js
47
species

node1158.members.0.js
3
species

node1159.members.0.js
39
species

node1160.members.0.js
268
9
genus

node1161.members.0.js
70
species

node1162.members.0.js
94
species

node1163.members.0.js
45
species

node1164.members.0.js
50
species

node1165.members.0.js
1006
24
genus

node1166.members.0.js
311
species

node1167.members.0.js
671
species

node1168.members.0.js
2363
215
family

3
genus

node1170.members.0.js
3
species

node1171.members.0.js
171
6
genus

node1172.members.0.js
105
species

node1173.members.0.js
60
species

305
genus

node1175.members.0.js
305
species

69
genus

node1177.members.0.js
69
species

node1178.members.0.js
731
117
genus

40
species

node1180.members.0.js
40
subspecies

node1181.members.0.js
125
species

node1182.members.0.js
57
species

node1183.members.0.js
97
species

node1184.members.0.js
295
species

80
genus

node1186.members.0.js
80
species

125
genus

node1188.members.0.js
125
species

node1189.members.0.js
434
194
genus

node1190.members.0.js
124
species

node1191.members.0.js
116
species

36
genus

node1193.members.0.js
36
species

194
genus

node1195.members.0.js
194
species

239
order

node1197.members.0.js
239
9
family

72
genus

node1199.members.0.js
46
species

node1200.members.0.js
26
species

41
genus

node1202.members.0.js
41
species

25
genus

node1204.members.0.js
25
species

39
genus

node1206.members.0.js
39
species

53
genus

node1208.members.0.js
53
species

3
genus

node1210.members.0.js
3
species

10
order

10
family

10
genus

node1214.members.0.js
10
species

12
order

12
family

9
genus

node1218.members.0.js
9
species

3
genus

node1220.members.0.js
3
species

node1221.members.0.js
33
species

140
order

67
family

17
genus

node1225.members.0.js
17
species

25
genus

node1227.members.0.js
25
species

25
genus

node1229.members.0.js
25
species

73
family

73
genus

node1232.members.0.js
52
species

node1233.members.0.js
3
species

node1234.members.0.js
18
species

10
order

10
family

node1237.members.0.js
10
6
genus

node1238.members.0.js
3
species

node1239.members.0.js
1
species

15
order

15
family

15
genus

node1243.members.0.js
15
species

57
phylum

21
class

21
order

21
family

21
genus

node1249.members.0.js
21
species

36
class

36
order

36
family

12
genus

node1254.members.0.js
12
species

20
genus

node1256.members.0.js
20
species

3
genus

node1258.members.0.js
3
species

1
genus

node1260.members.0.js
1
species

198
phylum

198
class

32
order

32
family

32
genus

node1266.members.0.js
25
species

node1267.members.0.js
7
species

node1268.members.0.js
155
10
order

33
family

node1270.members.0.js
33
3
genus

node1271.members.0.js
2
species

node1272.members.0.js
20
species

node1273.members.0.js
8
species

112
family

1
genus

node1276.members.0.js
1
species

node1277.members.0.js
109
43
genus

node1278.members.0.js
1
species

node1279.members.0.js
12
species

node1280.members.0.js
26
species

node1281.members.0.js
5
species

node1282.members.0.js
5
species

node1283.members.0.js
15
species

node1284.members.0.js
2
species

2
genus

node1286.members.0.js
2
species

11
family

9
genus

node1289.members.0.js
9
species

2
genus

node1291.members.0.js
2
species

node1292.members.0.js
9623
156
phylum

node1293.members.0.js
2258
2
class

47
order

1
family

1
genus

node1297.members.0.js
1
species

28
family

node1299.members.0.js
7
1
genus

node1300.members.0.js
6
species

8
genus

node1302.members.0.js
8
species

4
genus

4
species

node1305.members.0.js
4
subspecies

1
genus

node1307.members.0.js
1
species

8
genus

node1309.members.0.js
8
species

18
family

2
genus

node1312.members.0.js
2
species

16
genus

node1314.members.0.js
14
species

node1315.members.0.js
2
species

1
order

1
family

1
genus

node1319.members.0.js
1
species

node1320.members.0.js
2208
13
order

15
genus

node1322.members.0.js
15
species

20
family

12
genus

node1325.members.0.js
12
species

8
genus

node1327.members.0.js
5
species

node1328.members.0.js
3
species

11
family

11
genus

node1331.members.0.js
11
species

5
family

5
genus

node1334.members.0.js
5
species

469
family

27
genus

node1337.members.0.js
27
species

442
genus

node1339.members.0.js
442
species

121
family

116
genus

node1342.members.0.js
113
species

node1343.members.0.js
3
species

5
genus

node1345.members.0.js
5
species

91
family

3
genus

node1348.members.0.js
3
species

88
genus

node1350.members.0.js
87
species

node1351.members.0.js
1
species

node1352.members.0.js
1417
2
family

node1353.members.0.js
1413
527
genus

node1354.members.0.js
36
species

node1355.members.0.js
205
species

node1356.members.0.js
529
species

node1357.members.0.js
18
species

node1358.members.0.js
1
species

node1359.members.0.js
2
species

node1360.members.0.js
15
species

node1361.members.0.js
6
species

node1362.members.0.js
32
species

node1363.members.0.js
10
species

node1364.members.0.js
19
species

node1365.members.0.js
1
species

node1366.members.0.js
8
species

node1367.members.0.js
4
species

2
genus

node1369.members.0.js
2
species

5
family

5
genus

node1372.members.0.js
5
species

4
family

3
genus

node1375.members.0.js
1
species

node1376.members.0.js
2
species

1
genus

node1378.members.0.js
1
species

1
family

1
genus

node1381.members.0.js
1
species

13
family

13
genus

node1384.members.0.js
13
species

node1385.members.0.js
23
2
family

2
genus

node1387.members.0.js
2
species

2
genus

node1389.members.0.js
2
species

17
genus

node1391.members.0.js
17
species

node1392.members.0.js
7015
20
class

node1393.members.0.js
3163
3
order

19
family

19
genus

node1396.members.0.js
19
species

96
family

26
genus

node1399.members.0.js
26
species

node1400.members.0.js
70
1
genus

2
species

node1402.members.0.js
2
subspecies

node1403.members.0.js
3
2
species

node1404.members.0.js
1
subspecies

node1405.members.0.js
23
species

node1406.members.0.js
41
species

node1407.members.0.js
2682
15
family

node1408.members.0.js
1956
282
genus

node1409.members.0.js
1
species

node1410.members.0.js
22
species

node1411.members.0.js
36
species

node1412.members.0.js
265
species

1
species

node1414.members.0.js
1
subspecies

node1415.members.0.js
57
species

node1416.members.0.js
349
species

node1417.members.0.js
3
species

node1418.members.0.js
2
species

node1419.members.0.js
142
species

node1420.members.0.js
12
species

node1421.members.0.js
4
species

2
species

node1423.members.0.js
2
subspecies

node1424.members.0.js
233
species

node1425.members.0.js
367
species

node1426.members.0.js
35
species

node1427.members.0.js
47
species

node1428.members.0.js
31
species

node1429.members.0.js
3
species

node1430.members.0.js
1
species

node1431.members.0.js
5
species

node1432.members.0.js
45
species

node1433.members.0.js
9
species

2
species

node1435.members.0.js
2
subspecies

node1436.members.0.js
711
39
genus

node1437.members.0.js
672
29
species

node1438.members.0.js
560
subspecies

node1439.members.0.js
83
subspecies

node1440.members.0.js
91
2
family

76
genus

node1442.members.0.js
72
species

node1443.members.0.js
4
species

13
genus

node1445.members.0.js
13
species

node1446.members.0.js
272
1
family

node1447.members.0.js
269
73
genus

node1448.members.0.js
2
species

node1449.members.0.js
20
species

node1450.members.0.js
8
species

node1451.members.0.js
2
species

node1452.members.0.js
28
24
species

node1453.members.0.js
4
subspecies

node1454.members.0.js
2
species

node1455.members.0.js
8
species

node1456.members.0.js
16
species

node1457.members.0.js
2
species

node1458.members.0.js
62
species

node1459.members.0.js
17
species

node1460.members.0.js
1
species

node1461.members.0.js
2
species

26
species

node1463.members.0.js
26
subspecies

2
genus

node1465.members.0.js
1
species

node1466.members.0.js
1
species

node1467.members.0.js
3832
3
order

node1468.members.0.js
3301
6
family

37
genus

node1470.members.0.js
37
species

node1471.members.0.js
3258
304
genus

node1472.members.0.js
146
species

node1473.members.0.js
2322
species

node1474.members.0.js
134
117
species

node1475.members.0.js
17
subspecies

16
species

node1477.members.0.js
16
subspecies

node1478.members.0.js
161
species

node1479.members.0.js
75
species

node1480.members.0.js
15
species

node1481.members.0.js
4
species

51
species

node1483.members.0.js
51
subspecies

node1484.members.0.js
30
species

14
genus

node1486.members.0.js
1
species

node1487.members.0.js
13
species

9
family

9
genus

9
species

node1491.members.0.js
9
subspecies

38
family

2
genus

node1494.members.0.js
2
species

34
genus

node1496.members.0.js
2
species

node1497.members.0.js
1
species

node1498.members.0.js
16
species

1
species

node1500.members.0.js
1
subspecies

node1501.members.0.js
14
species

2
genus

node1503.members.0.js
2
species

5
family

5
genus

node1506.members.0.js
5
species

node1507.members.0.js
454
22
family

1
genus

node1509.members.0.js
1
species

2
genus

node1511.members.0.js
2
species

node1512.members.0.js
14
8
genus

node1513.members.0.js
4
species

node1514.members.0.js
2
species

1
genus

node1516.members.0.js
1
species

node1517.members.0.js
414
57
genus

node1518.members.0.js
21
species

node1519.members.0.js
16
species

node1520.members.0.js
1
species

node1521.members.0.js
4
species

node1522.members.0.js
4
species

node1523.members.0.js
6
species

node1524.members.0.js
5
species

node1525.members.0.js
4
species

node1526.members.0.js
270
species

node1527.members.0.js
26
species

8
family

node1529.members.0.js
8
6
genus

2
species

node1531.members.0.js
2
subspecies

189
class

189
order

10
family

node1535.members.0.js
10
8
genus

node1536.members.0.js
2
species

node1537.members.0.js
179
2
family

123
genus

node1539.members.0.js
123
species

1
genus

node1541.members.0.js
1
species

53
genus

node1543.members.0.js
53
species

5
class

5
order

5
family

1
genus

node1548.members.0.js
1
species

4
genus

node1550.members.0.js
4
species

64
phylum

22
class

3
order

3
family

3
genus

node1556.members.0.js
3
species

19
order

15
suborder

15
family

15
genus

node1561.members.0.js
8
species

node1562.members.0.js
7
species

4
suborder

4
family

node1565.members.0.js
4
3
genus

node1566.members.0.js
1
species

25
class

25
order

25
family

25
genus

node1571.members.0.js
25
species

1
class

1
order

1
family

1
genus

node1576.members.0.js
1
species

9
class

9
order

9
family

9
genus

node1581.members.0.js
9
species

7
class

1
order

1
family

1
genus

node1586.members.0.js
1
species

6
subclass

6
order

6
suborder

6
family

node1591.members.0.js
6
genus

5
phylum

5
class

5
order

5
family

4
genus

node1597.members.0.js
4
species

1
genus

node1599.members.0.js
1
species

134
superphylum

132
phylum

109
class

109
order

node1604.members.0.js
109
2
family

13
genus

node1606.members.0.js
13
species

14
genus

node1608.members.0.js
14
species

58
genus

node1610.members.0.js
29
species

node1611.members.0.js
29
species

node1612.members.0.js
22
2
genus

node1613.members.0.js
20
species

21
class

21
order

21
family

21
genus

node1618.members.0.js
21
species

2
genus

node1620.members.0.js
2
species

2
phylum

2
class

2
order

2
family

2
genus

node1626.members.0.js
2
species

7
phylum

7
class

7
order

7
family

3
genus

node1632.members.0.js
3
species

4
genus

node1634.members.0.js
4
species

1444
superphylum

1
phylum

1
class

1
order

1
family

1
genus

node1641.members.0.js
1
species

node1642.members.0.js
1402
11
phylum

9
order

9
family

9
genus

node1646.members.0.js
9
species

95
class

95
order

77
family

3
genus

node1651.members.0.js
3
species

9
genus

node1653.members.0.js
9
species

65
genus

node1655.members.0.js
65
species

14
family

3
genus

node1658.members.0.js
3
species

11
genus

node1660.members.0.js
11
species

4
family

2
genus

node1663.members.0.js
2
species

2
genus

node1665.members.0.js
2
species

546
class

node1667.members.0.js
546
44
order

16
family

node1669.members.0.js
16
2
genus

node1670.members.0.js
14
species

85
family

1
genus

node1673.members.0.js
1
species

17
genus

node1675.members.0.js
17
species

46
genus

node1677.members.0.js
20
species

node1678.members.0.js
26
species

10
genus

node1680.members.0.js
10
species

11
genus

node1682.members.0.js
11
species

16
genus

node1684.members.0.js
16
species

68
family

node1686.members.0.js
68
3
genus

node1687.members.0.js
53
species

node1688.members.0.js
4
species

node1689.members.0.js
8
species

317
family

317
genus

node1692.members.0.js
6
species

node1693.members.0.js
193
species

node1694.members.0.js
52
species

node1695.members.0.js
61
species

node1696.members.0.js
5
species

661
class

node1698.members.0.js
661
2
order

42
genus

node1700.members.0.js
42
species

node1701.members.0.js
606
6
family

1
genus

node1703.members.0.js
1
species

4
genus

node1705.members.0.js
4
species

1
genus

node1707.members.0.js
1
species

135
genus

node1709.members.0.js
135
species

6
genus

node1711.members.0.js
6
species

245
genus

node1713.members.0.js
9
species

node1714.members.0.js
82
species

node1715.members.0.js
147
species

node1716.members.0.js
1
species

node1717.members.0.js
6
species

node1718.members.0.js
23
species

7
genus

node1720.members.0.js
7
species

18
genus

node1722.members.0.js
18
species

19
genus

node1724.members.0.js
19
species

9
genus

node1726.members.0.js
9
species

4
genus

node1728.members.0.js
4
species

3
genus

node1730.members.0.js
3
species

79
genus

node1732.members.0.js
79
species

38
genus

node1734.members.0.js
38
species

8
genus

node1736.members.0.js
8
species

11
family

1
genus

node1739.members.0.js
1
species

10
genus

node1741.members.0.js
10
species

80
class

80
order

1
family

1
genus

node1746.members.0.js
1
species

75
family

16
genus

node1749.members.0.js
16
species

27
genus

node1751.members.0.js
27
species

8
genus

node1753.members.0.js
8
species

13
genus

node1755.members.0.js
13
species

7
genus

node1757.members.0.js
7
species

4
genus

node1759.members.0.js
4
species

4
family

4
genus

node1762.members.0.js
4
species

41
phylum

41
class

41
order

41
family

1
genus

node1768.members.0.js
1
species

3
genus

node1770.members.0.js
3
species

21
genus

node1772.members.0.js
18
species

node1773.members.0.js
3
species

15
genus

node1775.members.0.js
15
species

1
genus

node1777.members.0.js
1
species

374
phylum

node1779.members.0.js
374
2
class

31
order

31
family

12
genus

node1783.members.0.js
11
species

node1784.members.0.js
1
species

8
genus

node1786.members.0.js
4
species

node1787.members.0.js
3
species

node1788.members.0.js
1
species

6
genus

node1790.members.0.js
6
species

5
genus

node1792.members.0.js
5
species

node1793.members.0.js
341
1
order

10
family

10
genus

node1796.members.0.js
10
species

330
family

node1798.members.0.js
330
13
genus

node1799.members.0.js
53
species

node1800.members.0.js
1
species

node1801.members.0.js
220
species

node1802.members.0.js
12
species

node1803.members.0.js
13
species

node1804.members.0.js
17
species

node1805.members.0.js
1
species

2
phylum

2
class

2
order

2
family

2
genus

node1811.members.0.js
2
species

node1812.members.0.js
1598
21
superkingdom

node1813.members.0.js
7
species

22
phylum

1
order

1
family

1
genus

node1818.members.0.js
1
species

7
class

7
order

7
family

7
genus

node1823.members.0.js
7
species

14
order

14
family

node1826.members.0.js
14
genus

1
phylum

1
genus

node1829.members.0.js
1
species

40
phylum

40
class

node1832.members.0.js
37
21
order

5
family

5
genus

node1835.members.0.js
5
species

11
family

4
genus

node1838.members.0.js
4
species

node1839.members.0.js
7
genus

2
order

2
family

2
genus

node1843.members.0.js
2
species

1
order

1
family

1
genus

node1847.members.0.js
1
species

node1848.members.0.js
1507
113
phylum

839
class

node1850.members.0.js
6
2
order

4
family

4
genus

node1853.members.0.js
4
species

3
order

3
family

3
genus

node1857.members.0.js
3
species

node1858.members.0.js
830
33
order

247
family

node1860.members.0.js
247
10
genus

node1861.members.0.js
152
species

node1862.members.0.js
25
species

node1863.members.0.js
60
species

node1864.members.0.js
550
35
family

24
genus

node1866.members.0.js
10
species

node1867.members.0.js
6
species

node1868.members.0.js
8
species

24
genus

node1870.members.0.js
24
species

387
genus

node1872.members.0.js
387
species

80
genus

node1874.members.0.js
80
species

19
class

19
order

19
family

7
genus

node1879.members.0.js
7
species

node1880.members.0.js
12
2
genus

node1881.members.0.js
10
species

3
class

3
order

node1884.members.0.js
3
2
family

1
genus

node1886.members.0.js
1
species

19
class

19
order

19
family

node1890.members.0.js
19
17
genus

node1891.members.0.js
2
species

27
class

node1893.members.0.js
14
order

13
order

13
family

13
genus

node1897.members.0.js
13
species

207
class

50
order

50
family

12
genus

node1902.members.0.js
12
species

13
genus

node1904.members.0.js
13
species

8
genus

node1906.members.0.js
8
species

node1907.members.0.js
17
5
genus

node1908.members.0.js
7
species

node1909.members.0.js
5
species

128
order

node1911.members.0.js
128
4
family

1
genus

node1913.members.0.js
1
species

4
genus

node1915.members.0.js
4
species

5
genus

node1917.members.0.js
5
species

4
genus

node1919.members.0.js
4
species

4
genus

node1921.members.0.js
4
species

19
genus

node1923.members.0.js
19
species

87
genus

node1925.members.0.js
87
species

29
order

29
family

12
genus

node1929.members.0.js
7
species

node1930.members.0.js
5
species

1
genus

node1932.members.0.js
1
species

9
genus

node1934.members.0.js
2
species

node1935.members.0.js
7
species

7
genus

node1937.members.0.js
7
species

259
class

259
order

259
family

node1941.members.0.js
259
46
genus

node1942.members.0.js
2
species

node1943.members.0.js
190
species

node1944.members.0.js
2
species

node1945.members.0.js
19
species

node1946.members.0.js
21
genus
