# Supplementary figures and images for: Ultradeep Microbial Communities at 4.4 km within Crystalline Bedrock: Implications for Habitability in a Planetary Context
Source: Life (Basel). 2020 Jan 4;10(1):2. doi: 10.3390/life10010002 (PMC7175195; doi:10.3390/life10010002)

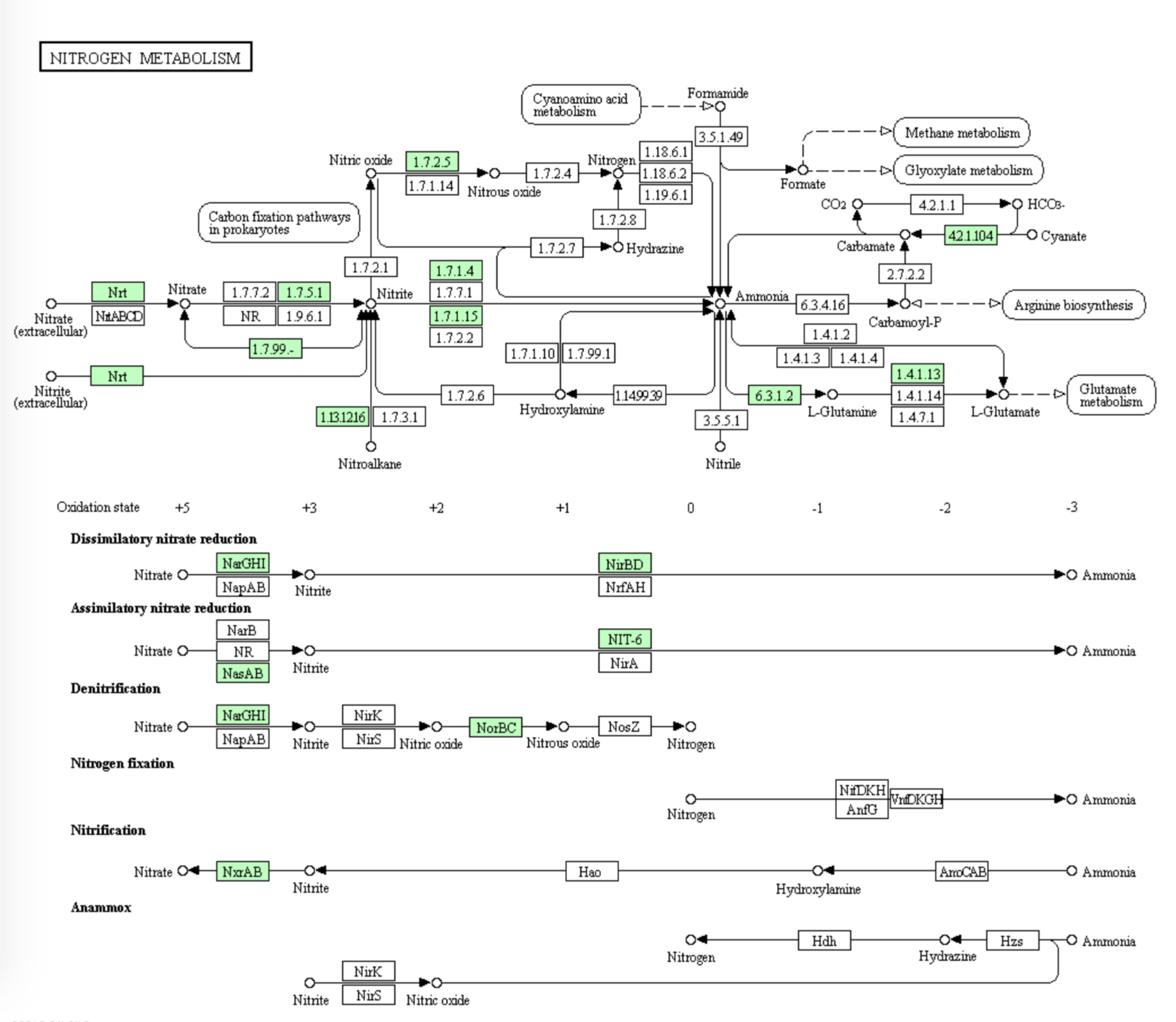

Supplement: Supplementary file 1 [file life-10-00002-s001.zip › SupplementaryFigure2_revised.png]
